# CSF1R inhibitor PLX5622 and environmental enrichment additively improve metabolic outcomes in middle-aged female mice

**DOI:** 10.18632/aging.102724

**Published:** 2020-02-02

**Authors:** Seemaab Ali, Anthony G. Mansour, Wei Huang, Nicholas J. Queen, Xiaokui Mo, Jacqueline M. Anderson, Quais N. Hassan II, Ripal S. Patel, Ryan K. Wilkins, Michael A. Caligiuri, Lei Cao

**Affiliations:** 1 Department of Cancer Biology and Genetics, College of Medicine, The Ohio State University, Columbus, OH 43210, USA; 2 The Ohio State University Comprehensive Cancer Center, The James Cancer Hospital and Solove Research Institute, Columbus, OH 43210, USA; 3 Medical Scientist Training Program, College of Medicine, The Ohio State University, Columbus, OH 43210, USA; 4 Department of Biomedical Informatics, College of Medicine, The Ohio State University, Columbus, OH 43210, USA; 5 Department of Hematological Malignancies and Stem Cell Transplantation, City of Hope National Medical Center and the Beckman Research Institute, Los Angeles, CA 91010, USA

**Keywords:** environmental enrichment, microglia, adipose, CSF1R, aging

## Abstract

As the elderly population grows, chronic metabolic dysfunction including obesity and diabetes are becoming increasingly common comorbidities. Hypothalamic inflammation through CNS resident microglia serves as a common pathway between developing obesity and developing systemic aging pathologies. Despite understanding aging as a life-long process involving interactions between individuals and their environment, limited studies address the dynamics of environment interactions with aging or aging therapeutics. We previously demonstrated environmental enrichment (EE) is an effective model for studying improved metabolic health and overall healthspan in mice, which acts through a brain-fat axis. Here we investigated the CSF1R inhibitor PLX5622 (PLX), which depletes microglia, and its effects on metabolic decline in aging in interaction with EE. PLX in combination with EE substantially improved metabolic outcomes in middle-aged female mice over PLX or EE alone. Chronic PLX treatment depleted 75% of microglia from the hypothalamus and reduced markers of inflammation without affecting brain-derived neurotrophic factor levels induced by EE. Adipose tissue remodeling and adipose tissue macrophage modulation were observed in response to CSF1R inhibition, which may contribute to the combined benefits seen in EE with PLX. Our study suggests benefits exist from combined drug and lifestyle interventions in aged animals.

## INTRODUCTION

The prevalence of obesity in the elderly is increasing, at the same time as the proportion of elderly in the population is on the rise [1]. Metabolic complications are an increasingly common comorbidity of aging [2]. Chronic metabolic diseases including cardiovascular disease, liver disease, and type 2 diabetes are major contributors to disability and mortality in the elderly, such that recent increases in lifespan are accompanied with increased rates of disability and no increase in overall healthspan [3].

Recently, attention has been focused on adipose tissue as an important player in the aging processes. Adipose tissue comprises a dynamic and interconnected endocrine organ responsive to the body’s needs for energy storage, nutrient sensing, and several immune functions. Aging is associated with a dysfunctional adipose phenotype of insulin resistance, glucose intolerance, and adipocyte cellular senescence [4]. This adipose tissue dysfunction during aging is thought to be a key driver of aging, leading to a systemic pro-inflammatory state and multi-organ dysfunction. This progression mirrors the pathologies associated with obesity [5]. The accumulation of macrophages into adipose tissue in states of obesity promotes a chronic pro-inflammatory microenvironment and adipose tissue dysfunction [6]. Because of the related pathophysiology of age-related systemic functional decline and obesity-associated diseases, and because of the increasing urgency for healthcare systems to address these problems as populations affected by them continue to grow, identifying and leveraging mechanisms for treatment is of considerable interest.

Weight and exercise interventions can improve physical function among overweight and obese older adults [7]. However, the dynamics of lifestyle interventions as they impact aging-related disease processes and interact with therapeutics for aging are poorly understood. Environmental enrichment (EE) is a model which allows us to study environment as a physiologically relevant manipulation of dysfunctional adipose tissue in order to identify and treat drivers of age-related metabolic decline. EE housing provides animals with species-appropriate cognitive, motor, sensory, and social stimuli with novelty and complexity. Compared to standard lab housing environments (SE), EE reduces age-related obesity, reduces hepatosteatosis, and improves glycemic control [8]. We previously identified a specific brain-adipose axis, the hypothalamic-sympathoneural-adipocyte (HSA) axis, through which EE-induced hypothalamic brain-derived neurotrophic factor (BDNF) expression leads to increased sympathetic tone to adipose tissue [9, 10]. Activation of the HSA axis results in the induction of a brown fat program in white adipose tissue, the suppression of leptin production and secretion, and an anti-obesity phenotype in middle-aged mice [8, 11, 12]. Importantly, adipose tissue response to sympathetic norepinephrine (NE) is impaired in old and obese mice by adipose tissue macrophages (ATMs) [13, 14].

Using the framework described, the hypothalamus is positioned as a central nervous system (CNS) link in aging and obesity. The hypothalamus is the central neuroendocrine system which regulates energy intake, consumption, and homeostasis. During both aging and the development of obesity, the hypothalamus chronically activates pro-inflammatory signals in what is termed “hypothalamic microinflammation” [15, 16]. Inflammation in the hypothalamus precedes the onset of obesity and diabetes, and evidence in both human and animal studies suggests that hypothalamic inflammation is causative in high fat diet- and lifestyle-induced metabolic syndrome [17, 18]. Recent evidence identifies the medial basal hypothalamus as coordinating systemic aging, including muscle weakness, reduced bone mass, and poor cognition, through neuroinflammation [19]. Microglia are the resident macrophages of the CNS and are the immune cells implicated in hypothalamic microinflammation. Microglia phagocytose cellular debris and initiate inflammatory responses to signal for peripheral immune cells to infiltrate the CNS. Microglia become dysfunctional during aging, developing a chronically inflamed state and becoming hyperreactive to immune challenges [20]. Chronic CNS cytokine exposure decreases overall BDNF expression in the brain [21]. However, microglia are also involved in learning-dependent synaptic plasticity through BDNF secretion [22]. With respect to EE, we reported that long-term EE affects microglial morphology, characterized as hypertrophy and ramification in the hypothalamus without increases in microglial cell density. These changes are accompanied with downregulation of inflammatory cytokine expression [12].

We were interested in the link between EE’s promotion of healthy aging – particularly metabolic improvement – and its modulation of microglial cells in female mice. Loss of microglia-derived BDNF impairs synaptic plasticity [22], but it is unknown whether microglia are essential for either the hypothalamic or the downstream metabolic outcomes associated with EE. Aged microglia are also hypothesized to decrease BDNF expression in the brain [23]. Therefore, their removal may instead improve baseline metabolism and improve outcomes after EE via the HSA axis. Thus, in this study, we used the colony stimulating factor 1 receptor (CSF1R) inhibitor PLX5622 (PLX) in order to understand the role of microglia in the metabolic improvement induced by EE. PLX5622 inhibits the receptor tyrosine kinase activity of CSF1R with high potency and selectivity [24, 25]. CSF1R receptor inhibitors deplete the CNS of microglia without causing behavioral or cognitive deficits [26, 27]. We treated animals with PLX5622, then housed animals in either SE or EE conditions and analyzed central and peripheral tissues to characterize the middle-aged mouse response to PLX5622 in combination with EE. We observed significant combined benefits of PLX treatment and EE housing on metabolic outcomes. EE modulated microglial gene expression and morphology as expected, while the depletion of microglia through PLX had no strong effects on the key gene expression signature of EE in the hypothalamus or on baseline BDNF. Peripherally, we observed significant changes in ATMs in response to PLX which may contribute to the combined benefits we observed.

## RESULTS

### Combined PLX5622 and environmental enrichment additively improve metabolic outcomes in middle-aged mice

**Figure 1:**
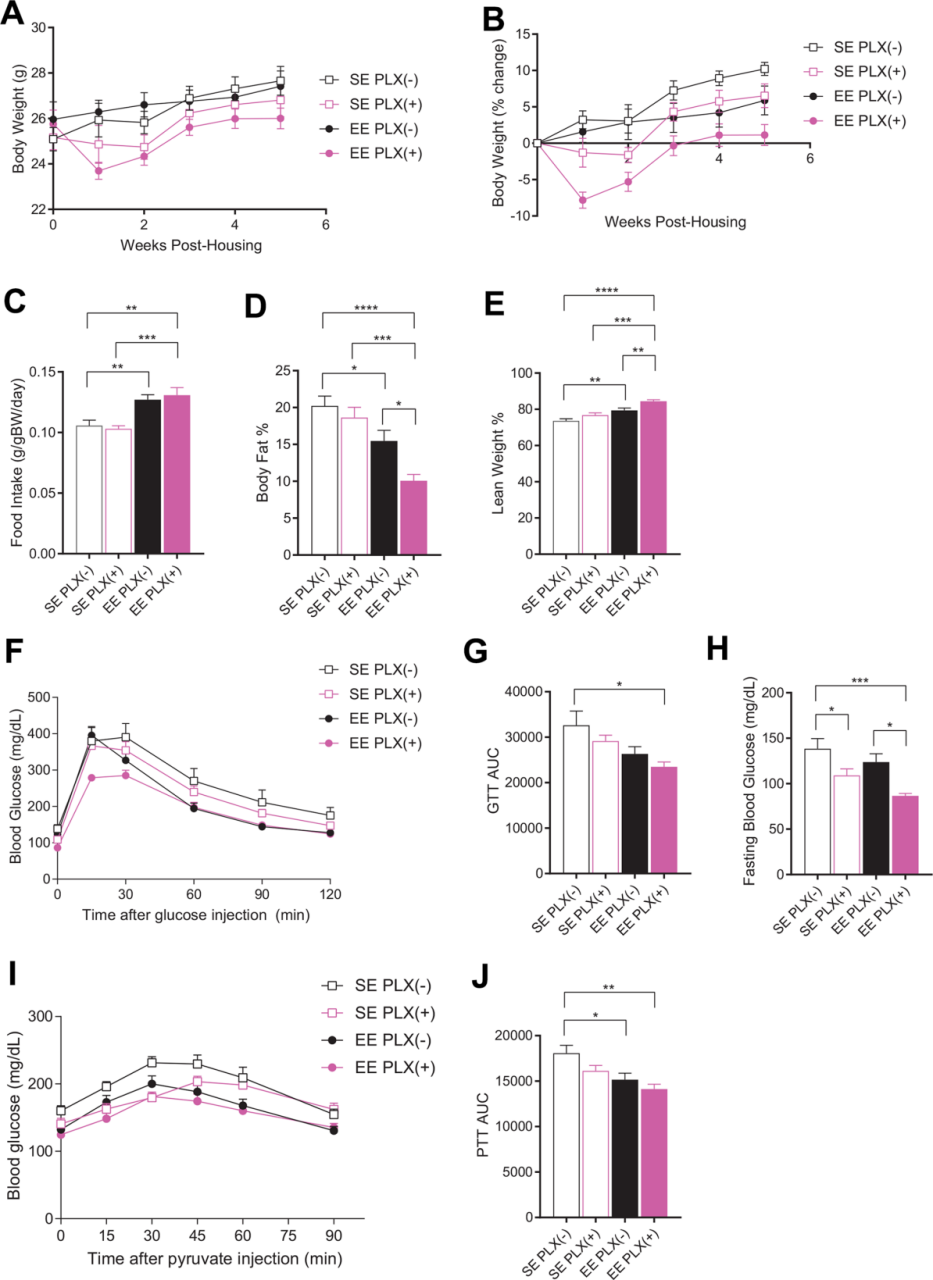
**Metabolic outcomes of PLX5622 and environmental enrichment in middle-aged mice.** (**A**) Body weights for animals on PLX(-) or PLX(+) diet in SE or EE across 5 weeks. (**B**) Body weight as a percentage change from starting body weight. (**C**) Food intake relative to body weight across 5 weeks. (**D**) Body fat proportion at 4 weeks. (**E**) Lean mass proportion. (**F**) Glucose tolerance test at 5 weeks. (**G**) Area under the curve. (**H**) Fasting blood glucose. (**I**) Pyruvate tolerance test at 6 weeks. (**J**) Area under the curve. (**A**, **B**, **D**–**J**) *n*=10 per group; (**C**) *n*=12, 2 cages per group across 6 weeks. *p<0.05, **p<0.01, ***p<0.001, ****p<0.0001. Values are means ± SEM. Statistical analyses are shown in Supplementary File 1.

Female mice aged 10-11 months were randomized to either SE or EE housing and initiated on either PLX5622 (PLX(+)) or normal chow diet (PLX(-)) 3 days before starting new housing conditions. Initially, animals on PLX showed considerable body weight loss after 1 week (Figure 1A, 1B). By 5 weeks of housing, animals on PLX and animals housed in EE both showed significantly reduced weight gain, with an additive effect in the combined EE PLX(+) group nearly preventing any body weight increase (Figure 1B). Food intake during this period also showed increased consumption across all EE housed animals, as has been described previously (Figure 1C) [8, 10]. PLX did not affect food intake in middle-aged mice, in contrast to its effect on mice on high fat diet [28]. Body composition measured by EchoMRI at 4 weeks showed main effects for both EE housing and PLX diet in reducing adiposity (Figure 1D). While PLX alone was not significantly different from SE PLX(-), the combination of EE PLX(+) dramatically reduced body fat to 10% of body weight. This level is comparable to young adult female mice (Supplementary Figure 1E). The increase of lean mass displayed similar results, with the greatest effect size in the EE PLX(+) combination group (Figure 1E).

Glucose tolerance in humans is an important indicator for frailty in old age [29]. Glucose tolerance measured at 5 weeks was significantly affected by housing, but not by PLX (Figure 1F, 1G). However, the combination treatment again showed the strongest effect and a significant reduction in glucose excursion compared to no treatment assessed by area under the curve analysis (Figure 1G.). Fasting blood glucose levels were lower in PLX(+) animals compared to PLX(-) counterparts, with a significant main effect for both PLX and EE observed. (Figure 1H). A pyruvate tolerance test serves as a measure of gluconeogenesis, primarily from the liver after fasting. We previously reported that middle-aged mice living in EE display lower blood glucose level during a pyruvate tolerance test compared to mice in SE [8]. Pyruvate tolerance measured at 6 weeks found reduced glucose excursions from both diet and housing (Figure 1I, 1J).

**Figure 2:**
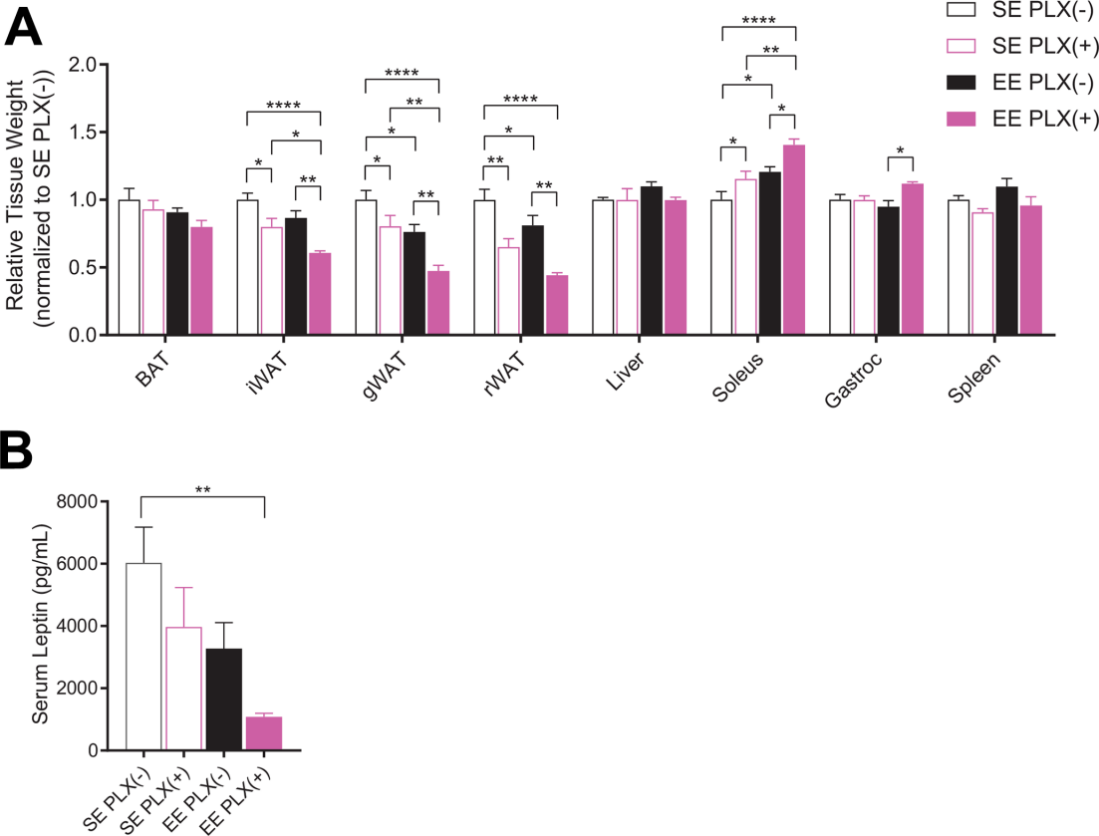
**Sacrifice measures of PLX5622 and environmental enrichment.** (**A**) Tissue weights normalized to body weight at sacrifice. (**B**) Serum leptin at sacrifice. (**A**, **B**) *n*=6 per group except gWAT, *n*=10 per group. *p<0.05, **p<0.01, ***p<0.001, ****p<0.0001. Values are means ± SEM. Statistical analyses are shown in Supplementary File 1.

At sacrifice, both PLX and EE alone reduced adipose depot mass normalized to body weight, including inguinal (iWAT), gonadal (gWAT) and retroperitoneal (rWAT) adipose depots (Figure 2A). The adipose depots were significantly further reduced in response to combined treatment. However, consistent with previous short-term EE studies, brown adipose tissue (BAT) was not significantly affected in middle-aged mice [8]. Relative soleus mass was increased in response to both treatments, but was significantly larger in EE PLX(+) than in either treatment alone. Spleen mass was significantly reduced in response to PLX, suggesting an effect of CSF1R inhibition in peripheral immune compartments. Serum leptin, secreted primarily by adipocytes, again displayed a pronounced combined effect of both PLX and EE treatments (Figure 2B).

To examine whether PLX’s metabolic effects were age-dependent, young adult mice were given PLX5622 diets and housed in SE. PLX trended to increase body weight after 2 months on diet (Supplementary Figure 1B). Otherwise, PLX showed no effect on food intake, body composition, or fasting blood glucose compared to normal chow (Supplementary Figure 1B–1G). Young mice on PLX showed reduced peak blood glucose, as well as reduced spleen size (Supplementary Figure 1F, 1H), similar to older mice (Figure 1F, Figure 2A).

### PLX5622 partially depletes microglial cells

**Figure 3:**
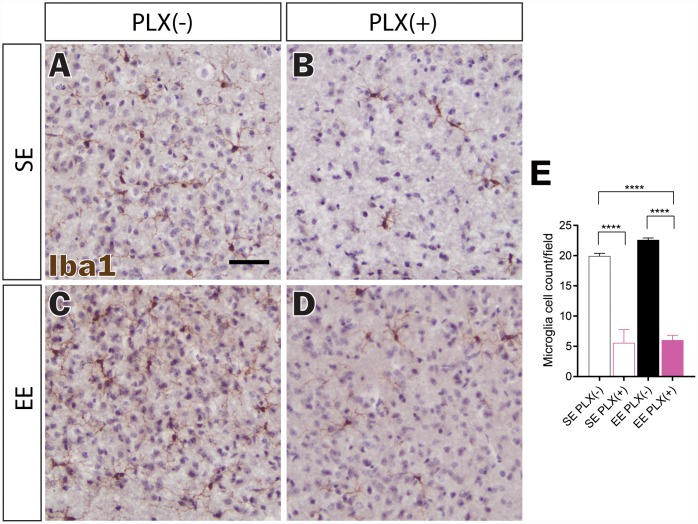
**Microglial depletion in response to PLX5622.** (**A**–**D**) Representative Iba1 immunohistochemistry of medial hypothalamus, in (**A**) SE PLX(-), (**B**) SE PLX(+), (**C**) EE PLX(-), and (**D**) EE PLX(+). (**E**) Microglia cell count within hypothalamus. Scale bar in A is 100 μm. (**E**) *n*=4 per group. ****p<0.0001. Values are means ± SEM. Statistical analyses are shown in Supplementary File 1.

In order to confirm central nervous system effects of PLX5622, Iba1 immunohistochemical staining was used to identify microglia (Figure 3A–3D). 3-day treatment of PLX5622 delivered in animal diets at 1200 mg/kg of chow has been reported to eliminate up to 95% of microglia from the hippocampus, and potentially 98% of hypothalamic microglia at 7 days [27, 30]. Pexidartinib (PLX3397), which acts similarly to PLX5622, has been reported to increasingly deplete microglia across 21 days of treatment, moving towards 99% clearance [26]. In our study, PLX treatment for 7 weeks reduced microglial density by nearly 75%, while not completely eliminating Iba+ cells within the hypothalamus (Figure 3E). Previously, we showed that EE does not affect overall Iba1+ cell count within the hypothalamus [12]. Furthermore, microglial hypertrophy and ramification were noted in response to long-term 8-month EE housing. EE housing for a shorter 7-week period also did not significantly affect microglial cell density (Figure 3E). This duration was sufficient to increase ramification morphologies for microglia in EE PLX(-) as well as for remaining microglia in EE PLX(+) (Figure 3C, 3D). Of note, EE housing did not significantly affect the number of remaining microglia in the hypothalamus in response to PLX.

### Hypothalamic gene expression

**Figure 4:**
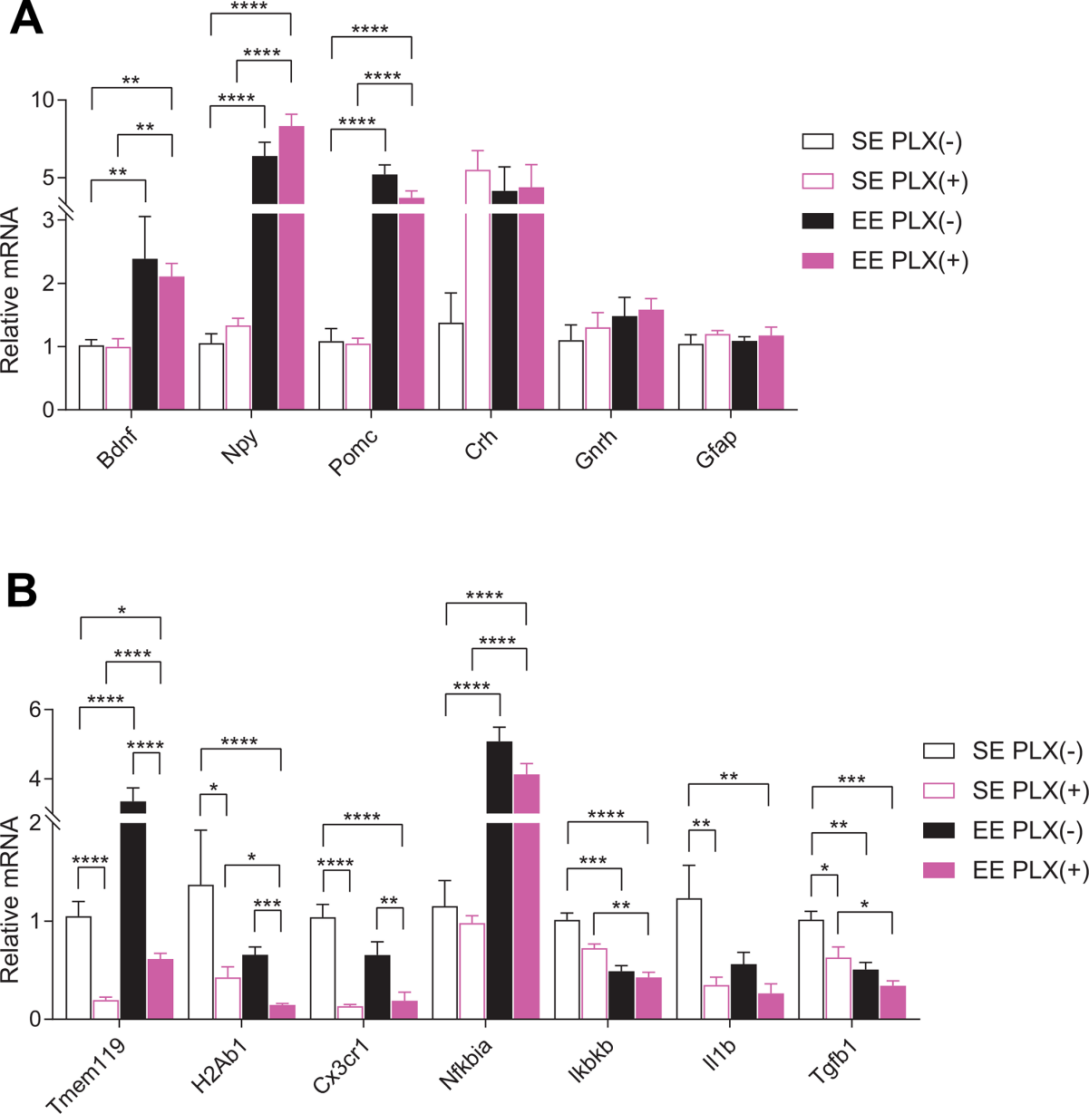
**Hypothalamic gene expression.** (**A**) Gene expression for hypothalamic and glia genes. (**B**) Microglial-associated, inflammatory pathway, and cytokine gene expression. (**A**–**B**) *n*=6 per group. *p<0.05, **p<0.01, ***p<0.001, ****p<0.0001. Values are means ± SEM. Statistical analyses are shown in Supplementary File 1.

Short-term EE paradigms have previously been shown to robustly increase the expression of *Bdnf* and several other feeding circuitry genes within the hypothalamus, including the orexigenic neuron marker neuropeptide Y (*Npy*) and anorexigenic proopiomelanocortin (*Pomc*) [8, 9]. To test whether these hypothalamic changes require the presence of microglia, we measured mRNA expression in the hypothalamus by RT-qPCR. The hypothalamic gene expression signature of EE, characterized as upregulation of *Bdnf*, *Npy*, and *Pomc*, was unaffected by PLX (Figure 4A). Housing, but not PLX, significantly increased *Bdnf* and *Pomc* expression. Orexigenic *Npy* mRNA was increased robustly by EE, but main effects of PLX on *Npy* also show increased expression. Stress hormone corticotropin releasing hormone (*Crh*), a key component of the hypothalamic-pituitary-adrenal (HPA) axis, trended to increasing in response to PLX. No effects were seen in gonadotropin releasing hormone (*Gnrh*) or the astrocytic marker glial fibrillary acidic protein (*Gfap*) by either treatment.

We next assessed microglial and inflammation-associated genes from the hypothalamus (Figure 4B). TMEM119 is a marker for CNS resident microglia which was significantly reduced in response to PLX, as expected. Consistent with our previous studies, *Tmem119* expression increased in response to EE without a concomitant microglia cell count increase [12]. This EE effect persisted in the presence of PLX, with *Tmem119* expression increasing approximately 3-fold in EE PLX(+) relative to SE PLX(+). We observed only a 40% reduction in *Tmem119* gene expression after PLX in combination with EE, relative to SE PLX(-), despite a 70% reduction in microglial cell count. By comparison, another microglial marker is fractalkine receptor CX3CR1, found specifically on microglia in the CNS. *Cx3cr1* expression was significantly reduced by PLX treatment but did not increase in response to EE.

Neuroinflammation throughout the brain develops progressively from middle to old age. In our study, young mice showed significantly lower gene expression of the pro-inflammatory interleukin 1β (*Il1b*) in the hypothalamus compared to middle aged mice (Supplementary Figure 2). We also observed lower levels of lymphocyte antigen 6 family member D (*Ly6d*) mRNA. *Ly6D* is expressed in certain lymphoid and dendritic cell immune populations not resident in the brain, which allows it to serve as a proxy for CNS immune trafficking. We have previously shown hypothalamic *Ly6D* expression is also reduced following long-term hypothalamic expression of BDNF [11]. Major histocompatibility complex class II (MHC II, encoded by *H2Ab1*) is highly expressed on primed microglia, which are abundant in aged brains [31, 32]. Depletion and repopulation of microglia in the aged brain following PLX5622 exposure is insufficient to change MHC II expression on recovered microglia [33]. Following both EE and PLX exposure, *H2Ab1* decreased in the hypothalamus. EE PLX(+) *H2Ab1* expression was significantly decreased below the levels seen in SE PLX(+), which represents a combined effect of EE on the state of microglia remaining in the hypothalamus following PLX treatment. The NFκB inflammatory signaling activator, inhibitor of NFκB kinase subunit β (*Ikbkb*), and the immunosuppressive cytokine transforming growth factor β 1 (*Tgfb1*) were both reduced in response to both treatments. *Il1b* was also significantly reduced in response to PLX. Inhibitor of NFκB α (*Nfkbia*), which provides inhibitory feedback to NFκB pathway activation, was strongly upregulated in response to EE and not affected by PLX.

Together, these findings show that EE strongly affects hypothalamic microglia gene expression in complex ways without changing the number of microglia in the hypothalamus. Partial depletion of microglia, however, has no effect on EE-induced hypothalamic gene expression central to the HSA axis.

### Adipose tissue macrophages

**Figure 5:**
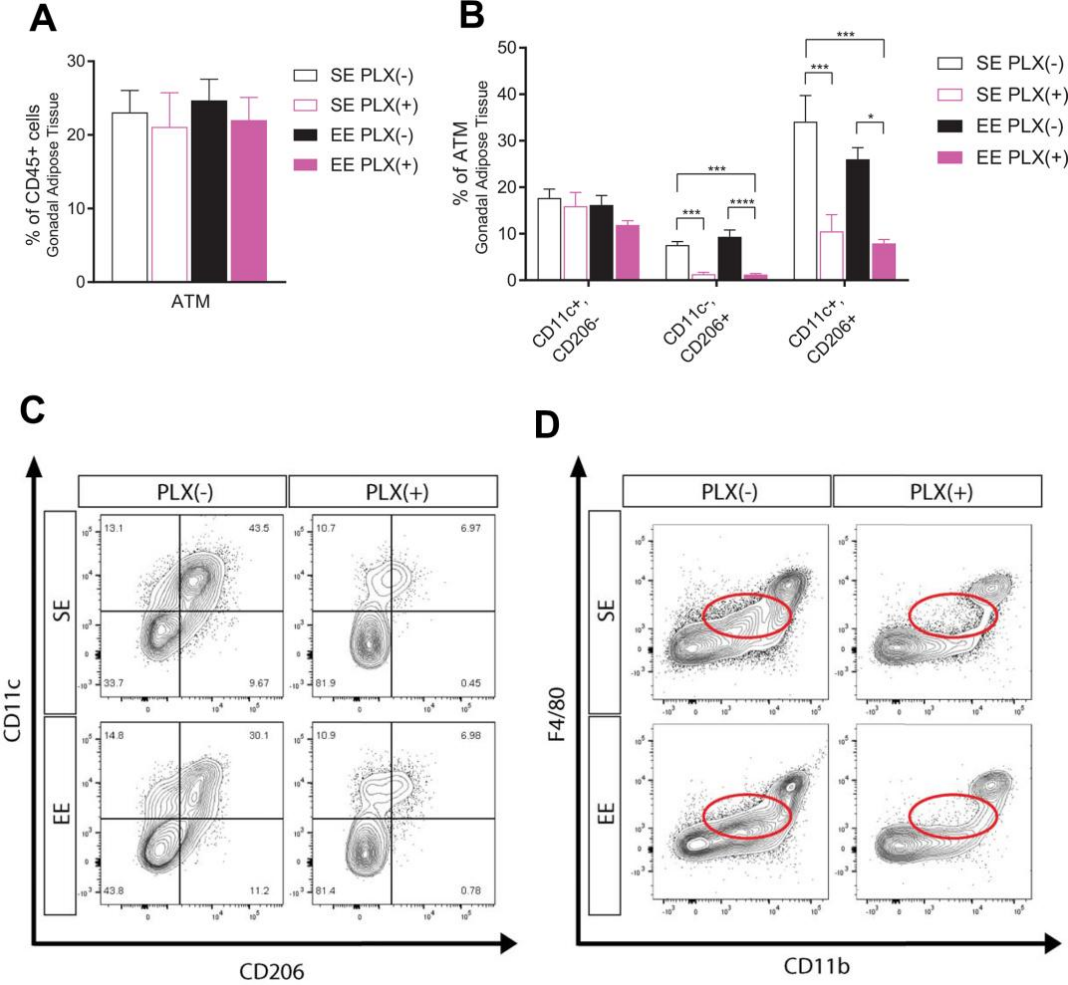
**Adipose tissue response to PLX5622 and environmental enrichment in middle-aged mice.** (**A**) Adipose tissue macrophages (ATM) from gonadal white adipose tissue. (**B**) ATM polarization, including CD11c+, CD206+ cells. (**C**) Representative flow diagram of ATM CD206+ subset depletion. (**D**) Representative flow diagram of F4/80^low^, CD11b^low^ population depletion. (**A**–**C**) *n*=5-6 per group. *p<0.05, **p<0.01, ***p<0.001, ****p<0.0001. Values are means ± SEM. Statistical analyses are shown in Supplementary File 1.

In addition to CNS microglia modulation, peripheral immune responses to both treatments potentially play a role in the metabolic outcomes we observed [34]. ATMs contribute to the development and maintenance of obese states, and age-related changes in these macrophages prevent fat loss and lipolysis [13, 14, 35, 36]. Specifically, aged ATMs uptake and metabolize catecholamines, which inhibits SNS signals to adipose tissue such as NE-induced lipolysis of adipocytes. To investigate ATMs, we isolated stromal vascular fraction (SVF) from gWAT and identified macrophages by flow cytometry. ATMs were defined as F4/80+, CD11b+ cells as a subset of CD45+, CD19- live cells (Supplementary Figure 3). Macrophages are plastic cells whose phenotype can change in response to microenvironment conditions. Classically activated “M1” macrophage accumulate in adipose tissue in obesity states and are associated with insulin resistance and high levels of pro-inflammatory cytokines, while alternatively activated “M2” macrophages are present in leaner adipose tissue [36]. The surface marker CD11c was used to identify M1 macrophages, while CD206 was used to identify M2. As a subset of CD19- immune cells, ATM percentage was unaffected by either treatment class despite the significant reduction in adipose tissue between groups (Figure 5A). Of macrophage subpopulations, the largest proportion of macrophages we identified in PLX(-) animals was CD11c+, CD206+. These macrophages increase in response to fasting, which induces lipolysis in WAT through NE release by the SNS, in young wild type (WT) and aged Nlrp3 -/- mice but not aged WT mice [13]. In humans, ATMs of this marker set have been identified as sources of proinflammatory cytokines and drivers of insulin resistance, with a mix of M1 and M2 features [37]. Surprisingly, while CD11c+, CD206- M1 ATMs were unchanged in response to PLX, both CD11c-, CD206+ and CD11c+, CD206+ ATMs were significantly depleted (Figure 5B). Representative plots show CD206+ cells regardless of CD11c status as nearly eliminated, with a small population of CD11c+, CD206+ ATMs remaining (Figure 5C). This study also identified a subpopulation of F4/80^low^, CD11b^low^ immune cells which were eliminated by PLX treatment, but were not the primary ATM population (Figure 5D). EE did not result in ATM population shifts despite producing leaner middle-aged mice.

### Adipose gene expression

**Figure 6:**
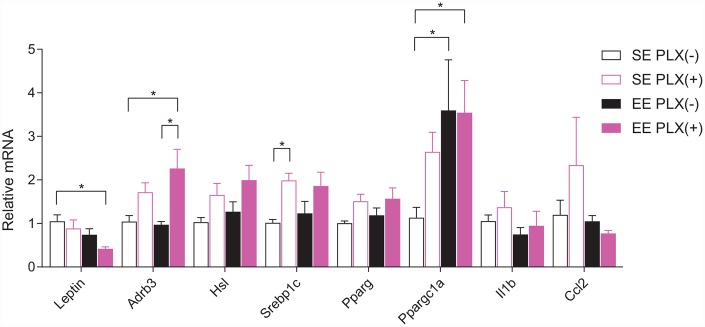
**Retroperitoneal white adipose tissue gene expression.** *n*=5-6 per group. *p<0.05, **p<0.01, ***p<0.001, ****p<0.0001. Values are means ± SEM. Statistical analyses are shown in Supplementary File 1.

We next examined rWAT for the adipose gene expression signature associated with EE, in order to identify changes in adipose tissue response following PLX treatment that may account for the combined treatment effect [10]. In contrast to gWAT, rWAT mass was reduced to a greater extent by PLX than by EE (Figure 2A). RT-qPCR of bulk rWAT showed significant PLX effects across several genes involved with sympathetic response (Figure 6). *Lep* expression from rWAT displayed a similar trend to overall adiposity and was consistent with circulating leptin. β3-adrenergic receptors (*Adrb3*) are the responsive receptor to sympathetic norepinephrine release onto adipose tissue. Main effects of PLX treatment show increased expression of *Adrb3* mRNA in rWAT. Hormone sensitive lipase (*Hsl*), which mobilizes lipids in response to sympathetic tone, also shows a main effect of increased in expression in PLX(+) animals. Lipogenic sterol regulatory element-binding transcription factor 1c (*Srebp1c*) and peroxisome proliferator-activated receptor γ (*Pparg*) showed significant main effects of PLX, also increasing in response to PLX treatment. PPARγ coactivator 1α (encoded by *Ppargc1a*), which induces mitochondrial biogenesis, was increased by both EE and PLX. Inflammatory cytokine *Il1b* and monocyte chemokine *Ccl2* did not display significant trends. Overall, PLX-responsive adipose tissue displayed gene expression trends consistent with sympathetic nervous system (SNS) action on adipose tissue.

## DISCUSSION

Our study investigated the combined effects of CSF1R inhibition and enriched environments on age-related metabolic decline and resulted in several interesting findings. 1) PLX5622 treatment exerted some limited metabolic benefits in middle-aged mice but not young adults. If PLX5622 acted through microglial removal alone, these data are in support of the hypothesis that aged microglia may contribute to age-related metabolic decline [15, 16]. However, our new evidence suggests that PLX5622 may also improve metabolism through peripheral immune modulation. 2) The beneficial metabolic effects of EE were not dependent on microglia, as their depletion by PLX did not attenuate EE effects or affect hypothalamic levels of BDNF. 3) EE and longitudinal PLX act together to reduce adiposity and improve glucose tolerance, in cases where PLX alone and EE alone have weaker effects or no effect. If this combined effect is primarily mediated by CNS changes, two potential hypotheses emerge: First, aged microglia in the hypothalamus might be responsible for local inhibition of EE-induced signals downstream of or parallel to BDNF. If so, then without affecting BDNF levels (Figure 4A), microglial removal would allow full display of EE metabolic benefits. Alternatively, our results also show both reduced hypothalamic inflammatory gene expression in EE and incomplete microglial depletion to PLX alone. Therefore, EE may improve the function of the remaining microglia in PLX-treated mice and thereby further alleviate aging-related metabolic decline. 4) The combined metabolic benefit of PLX5622 and EE may instead be peripherally driven. EE is established as a multifaceted environmental intervention which produces changes across many body systems, including in the CNS and in adipose tissue. However, PLX5622 has primarily been described as potently affecting CNS macrophages, with significantly smaller peripheral immune effects [28, 38, 39]. It is possible that the combined metabolic benefit of drug and environment involves peripheral effects – not only in the adipose tissue, but also other organs such as liver and muscle.

In order to identify the mechanisms by which EE acts together with PLX5622 to enhance adiposity reduction and improve glycemic control, we investigated both the CNS and adipose tissue. In the CNS, EE acted to change microglial morphology and microglial gene expression in middle-aged mice. These findings appear consistent with long-term EE housing and long-term viral expression of BDNF in middle-aged mice [8, 11, 12]. Several of these effects remained or were compounded in response to CSF1R inhibition and depletion of microglia, suggesting that the stimulus behind hypothalamic changes causing microglial responses remains in the presence of PLX for remaining microglia. A major candidate for this stimulus is BDNF, the central coordinator for many CNS and peripheral effects of EE. We previously demonstrated that neuronal overexpression of BDNF reproduces various effects of EE including systemic metabolism, adipose remodeling, immune modulations, cancer, and aging [9–11, 40–42].

For consideration, the opposite direction of this association of microglia in response to EE or BDNF does not bear out: baseline *Bdnf* expression was unaffected by microglial depletion. Therefore, in middle age, microglia are likely not diminishing *Bdnf* expression in the hypothalamus. Additionally, microglia appear not to be essential for the metabolic changes associated with EE or to be a large source of the *Bdnf* mRNA signature of EE in the hypothalamus. While inflammatory cytokines such as IL-1β were reduced in response to PLX, no changes were observed in *Bdnf* expression in response to PLX, with or without EE. This indicates that drug-induced reductions in microglia and in age-related elevated CNS cytokine levels were not a significant modulator of BDNF. This study supports the notion that neuronal BDNF acts as the key mediator of the changes we observe in EE. Other glial and endothelial cell sources are not ruled out here. Based on these observations, we propose that neuronal BDNF signaling mediates EE-induced changes in microglia. Investigations on this hypothesis are currently underway in our lab.

In adipose tissue, our data suggest that PLX treatment in middle-aged animals promoted a sympathetic-sensitive phenotype. Chronic sympathetic overactivity is a shared hallmark of obesity and aging [43]. SNS activation happens in response to elevated leptin and lipid signals in obese states, but chronic SNS activation desensitizes β-adrenergic signaling in adipocytes [44]. Inflammasome activated ATMs in older mice display upregulated catecholamine catabolism and block lipolysis signals from the SNS [13]. Under PLX treatment, the ATM phenotypic shifts we observed were associated with increased sympathetic responsiveness markers. Other studies using CSF1R inhibitors to limit the development or progression of obesity show some limited effects, which are primarily explained by changes in the hypothalamus [28, 30]. On the other hand, a study investigating ATMs in animals on a HFD in response to pexidartinib showed no benefit of drug treatment alone, despite substantial changes observed in the macrophage compartment within adipose tissue [45]. This observation is somewhat congruent with our data, which show no benefit by PLX5622 alone in glucose tolerance and whole-body adiposity (Figure 1D, 1F, 1G). However, we do observe reductions in single adipose tissue depots following PLX5622. Differences between our middle-aged mouse model and the young adult HFD mouse model given pexidartinib may explain SE differences between the studies: young HFD-fed mice may not have significantly altered sympathetic nerve or macrophage function in adipose tissue contributing to their metabolic disturbance. Removing these ATMs would therefore not provide any benefit. We further observe significant combined benefits of PLX with EE, which is known to act through the SNS to induce lean animal phenotypes. Together, our findings support the hypothesis of sympathetic blockade of adipose tissue contributing to the development of obesity and metabolic dysfunction during aging, which is amenable to certain treatments acting on sympathetic efficacy.

Pathway-overlapping interactions between therapeutics are increasingly important to identify. Recently, the widely recommended dual first-line treatment modalities of metformin and exercise intervention for diabetes are coming under scrutiny as antagonistic treatments [46–48]. Metformin has otherwise appeared individually promising for mitigating aging-related functional decline and improving longevity [49, 50]. Lifestyle changes like increased exercise are also always recommended treatments for patients with metabolic syndrome. The combination of these prescriptions was a foreseeable and addressable problem for biomedical and clinical research. Aging therapeutics being developed must be amenable to implementation in real patient populations. To that end, the EE model provides insight into biological pathways that are likely at play in patient populations, but are hidden in SE housed laboratory animals. Thus, identifying drugs that have effects on top of or in combination with EE pathways then becomes highly relevant for translatability.

A major concern facing the use of microglia-depleting drugs as long-term therapies is reducing the capacity of debris clearance and synaptic maintenance in the healthy brain. As of yet, it is not clear that these drugs deplete microglia in humans. Our data suggest that, while hypothalamic targeting of CSF1R antagonists has its benefits, peripheral targets like adipose tissue with directed delivery or reformulation to prevent blood-brain barrier crossing can also potentially be effective. Short-term treatments like diet, exercise, or other lifestyle interventions coinciding with a short-term course of PLX drugs could also be therapeutic, based on our data, while mitigating the harms of chronic drug treatment. Still other strategies being investigated include “refreshing” microglia or macrophages depleted by CSF1R antagonists and then allowing repopulation after a short treatment course [33, 51].

One limitation of this study is being unable to distinguish the central microglia and peripheral macrophage effects of PLX5622. PLX5622 is frequently cited as a CSF1R inhibitor which specifically depletes microglia from the CNS without affecting peripheral monocyte and macrophage populations [28, 38, 39]. In this study, we observed several clear peripheral immune changes in response to PLX5622, including in the spleen (Supplementary Figures 4, 5) and adipose tissue (Figure 5) which were previously unidentified. PLX5622 treatment also does not impair the chemotactic potential of peripheral myeloid cells to infiltrate into either the CNS or adipose tissue [28, 52]. Therefore, the phenotypic changes to these cells we identified are likely to be highly important for the functional significance of these cells. On the other hand, we cannot rule out that these peripheral immune changes we observed are not downstream of microglia depletion in the aged mice. Future studies using inducible genetic depletion of microglia and macrophages will delineate the central and peripheral immune modulations and their roles in aging-related metabolic decline in the context of drug and lifestyle interventions.

In summary, we demonstrated that PLX-induced microglial removal significantly affects measures of age-related metabolic decline. This effect is limited with drug treatment alone, but EE housing with PLX results in a robust drug-environment combined effect which was previously undescribed. We further identified CNS and adipose tissue changes induced by both treatments which may contribute to the observed phenotype, including reduction of hypothalamic inflammatory signals and generating a sympathetic-sensitive pattern in adipose tissue. Multi-modal interactive studies like this one will continue to provide insight into novel, physiologic, and biological mechanisms not otherwise observed with standard laboratory housing, which hopefully can inform future therapeutic approaches.

## MATERIALS AND METHODS

### Animals and housing

Female 10- to 11-month old middle-aged C57BL/6 mice from the National Institute on Aging Aged Rodent Colonies were randomized to live in either EE or SE conditions for 7 weeks. For SE, mice were housed in standard mouse cage of 30.5 cm x 17 cm x 15 cm (5 mice per cage). For EE, mice were housed in large cages (63 cm x 49 cm x 44 cm, 5 mice per cage) supplemented with running wheels, tunnels, igloos, huts, retreats, wood toys, and a maze in addition to chow and water. In young mouse studies, 2-month old young adult female C57BL/6 mice from Charles River were housed in SE for 8 weeks. Mice were housed in a 12:12 light:dark cycle, in temperature (22-23 °C)- and humidity-controlled rooms with access to food and water *ad libitum*. All animal experiments were carried out in compliance with the regulations of the Ohio State University Institutional Animal Care and Use Committee.

Males were not used in this study. Adult and aged males will respond to randomization into new housing groups with increased and sometimes serious fighting while establishing new social structures, requiring removal of and occasionally euthanasia for study subjects. In response to new and enriched environments, males also change in behavior and have been reported to increase territorial behavior and aggression.

### Diet

CSF1R inhibitor PLX5622 was provided by Plexxikon, Inc. (Berkley, CA) and formulated at 1200 ppm into rodent diet AIN-76A (12% fat, caloric density 3.86kcal/g) by Research Diets, Inc. (New Brunswick, NJ). Animals received either the PLX5622 formulated diet or a normal chow diet (AIN-76A) 3 days prior to initiating housing.

### Body weight and food intake

Body weight and food intake were monitored weekly. Food intake was averaged per mouse per week in each cage.

### Body composition

EchoMRI was used to measure body composition of fat, lean, free water, and total water masses in live mice without anesthesia. EchoMRI imaging was performed with the EchoMRI Analyzer at the Small Animal Imaging Core of The Dorothy M. Davis Heart & Lung Research Institute, Ohio State University.

### Glucose tolerance test

After an overnight fast (>16 hr), mice were injected intraperitoneally with a 20% glucose solution (2 g/kg body weight). Blood was obtained from the tail before injection and at 15, 30, 60, 90, and 120 min after glucose injection. Blood glucose concentrations were measured with a portable glucose meter (Bayer Contour Next, Parsippany, NJ).

### Pyruvate tolerance test

Mice were injected intraperitoneally with a 15% sodium pyruvate solution (1.5 g sodium pyruvate per kg body weight) after an overnight fast. Blood was obtained from the tail before injection and at 15, 30, 45, 60, and 90 min after sodium pyruvate injection. Blood glucose concentrations were measured with a portable glucose meter (Bayer Contour Next, Parsippany, NJ).

### Tissue harvest

At sacrifice, animals were anesthetized by isoflurane and decapitated. Truncal blood was harvested at euthanasia and serum collected. Serum was diluted at least 1:5 in serum assay diluent and assayed for Leptin using the DuoSet ELISA Development System (R&D Systems, Minneapolis, MN). Brown adipose tissue, gonadal, inguinal, and retroperitoneal white adipose tissue, liver, soleus, gastrocnemius, spleen, and hypothalamus were dissected and weighed.

### Perfusion

A subset of mice was anesthetized and transcardially perfused with PBS, followed by 4% paraformaldehyde (PFA) (Sigma, St. Louis, MO) in PBS. Fixed brains were extracted and incubated in 4% PFA on a rocker overnight at 4°C. Brains were then rinsed of PFA in PBS before being submerged in 30% sucrose in PBS with 0.03% sodium azide for at least 3 days at 4°C.

### Immunohistochemistry

Fixed brains were sectioned into 30-μm free-floating slices on a ThermoScientific HM525NX cryostat (Waltham, MA), and subjected to citrate-based antigen retrieval followed by incubation with rabbit anti-Iba1 (FUJIFILM Wako Chemicals USA, Cat. No.019-19741, Richmond, VA, 1:1000) overnight at 4°C. The sections were visualized with DAB and counterstained with hematoxylin. Microscopy was performed on an Olympus BX43 light microscope (Olympus Corporation, Center Valley, PA). For each animal, 2 sections were sampled between -1.0 and -1.8 mm from bregma, and 6 medial hypothalamus fields were systematically captured for each section. Manual cell counts were averaged across 12 fields for each animal.

### Quantitative RT-qPCR

Hypothalamus and rWAT were dissected at sacrifice. RNA was isolated using Qiagen RNeasy Mini kit with RNase-free DNase treatment (Germantown, MD), and first-strand cDNA was reverse transcribed using TaqMan Reverse Transcription Reagents (Applied Biosystems, Foster City, CA). We performed quantitative PCR on a StepOnePlus Real-Time PCR System (Applied Biosystems) with Power SYBR Green PCR Master Mix (Applied Biosystems). Data were calibrated to endogenous control Hprt1 for hypothalamus and Actinb for rWAT. Relative relative gene expression was quantified using the 2^-ΔΔCT^ method. Our primer sequences are available on request.

### Stromal vascular fraction

As previously described [53], dissected gonadal white adipose tissues were transferred to a 12-well culture plate containing Krebs-Ringer HEPES buffer, then minced to a fine consistency. Collagenase II (MilliporeSigma, St. Louis, MO) at 1.2 mg/mL was added to each well, and mixtures were incubated at 37°C with shaking at 90 RPM for 45 min. Mixtures were then passed through a 100-μm strainer and spun. Pelleted stromal vascular fraction cells were collected, treated with RBC lysis ammonium chloride solution for 5 min, washed, and counted.

### Splenocyte isolation

Dissected spleens were mechanically dissociated through a 70-μm strainer to obtain single cell suspension. RBCs were lysed with ammonium chloride solution, and then splenocytes were washed and re-suspended in FACS buffer. Cells were counted using the Cellometer Auto 2000 (Nexcelom Bioscience, Lawrence, MA).

### Flow cytometry

For surface staining, cells were stained with a fluorescent dye conjugated antibody with the appropriate surface markers for 20 min. The antibodies used for flow cytometry immunophenotyping are listed in Supplementary Table 1. Cell events were acquired using an LSRII flow cytometer (BD Biosciences, San Jose, CA) and the results were analyzed using FlowJo v10 (FlowJo, LLC, Ashland, OR).

### Statistical analysis

Data are expressed as mean ± SEM and significance was set at *p*<0.05. We used GraphPad Prism v7.00 (GraphPad, La Jolla, CA) and SPSS Statistics v25 (IBM, Armonk, NY) to analyze each data set. Two-way ANOVA was used to make comparisons between drug treatment and housing condition combinations. Post tests were performed for relevant comparisons using a Holm-Sidak multiple comparison correction. Student’s *t*-tests for direct comparisons and two-way repeated measures ANOVA for time course data were used for young mouse tests between drug treatment groups. Statistical summary is shown in Supplementary File 1.

## Supplementary Material

Supplementary Figures

Supplementary Table 1

Supplementary File 1
