# Supplementary material for: CSF1R inhibitor PLX5622 and environmental enrichment additively improve metabolic outcomes in middle-aged female mice
Source: Aging (Albany NY). 2020 Feb 2;12(3):2101–22. doi: 10.18632/aging.102724 (PMC7041757; doi:10.18632/aging.102724)
Supplement: Supplementary Figures [file aging-12-102724-s002..pdf]

SUPPLEMENTARY FIGURES

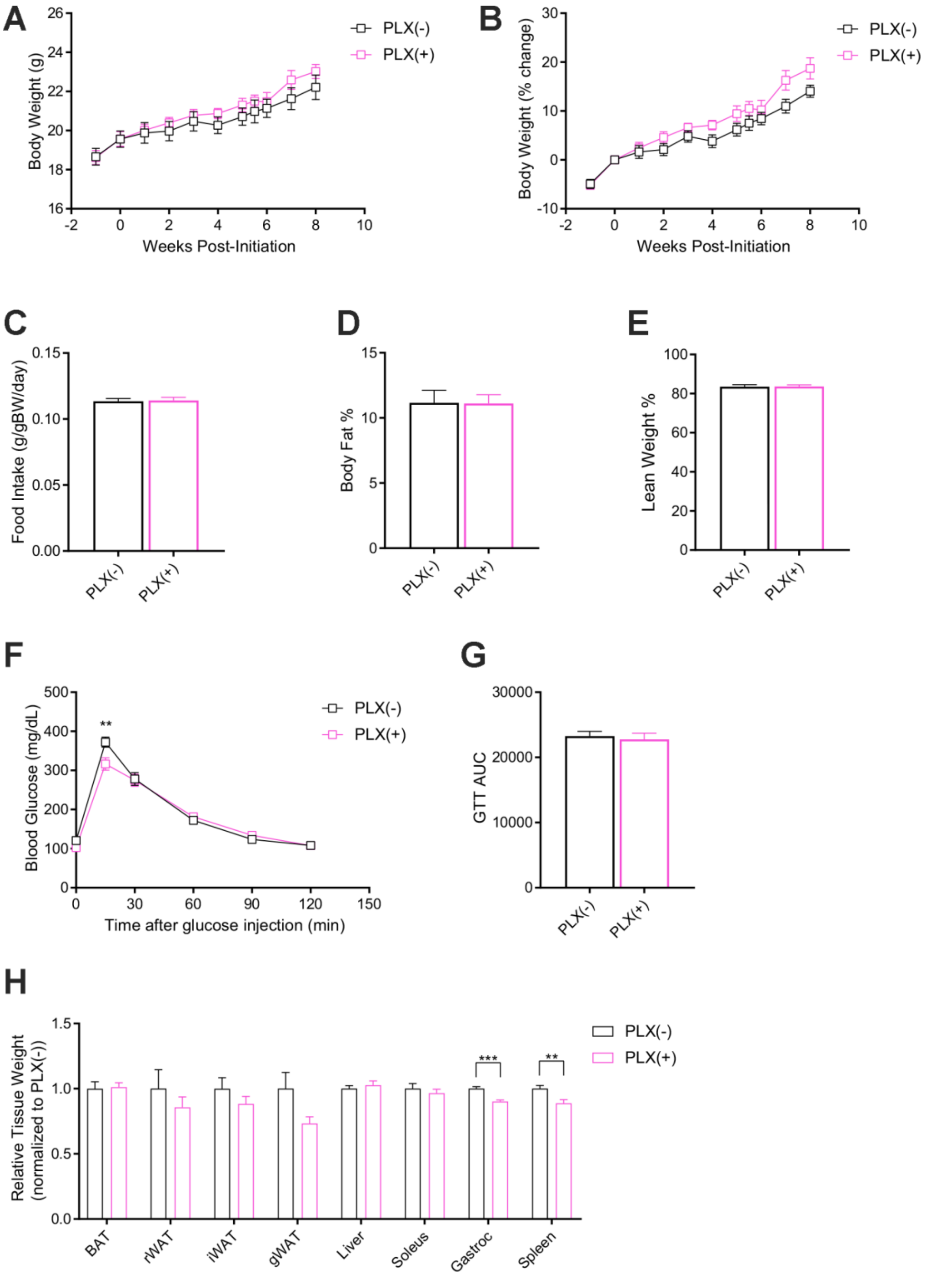

Supplementary Figure 1. Metabolic outcomes of PLX5622 in young mice. (**A**) Body weights for animals on PLX(-) or PLX(+) diets across 8 weeks. (**B**) Body weight as a percentage change from study starting body weight. (**C**) Body weight normalized food intake across 8 weeks. (**D**) Body fat mass percent at 5 weeks. (**E**) Lean mass percent. (**F**) Glucose tolerance test at 6 weeks. (**G**) Area under the curve. (**H**) Relative tissue weight at sacrifice. (**A**, **B**, **D**–**G**) *n*=10 per group, (**C**) n=14, 2 cages per group across 8 weeks. (**H**) *n*=7 per group. ***p*<0.01, ****p*<0.001. Values are means ± SEM. Statistical analyses are shown in Supplementary File 1.

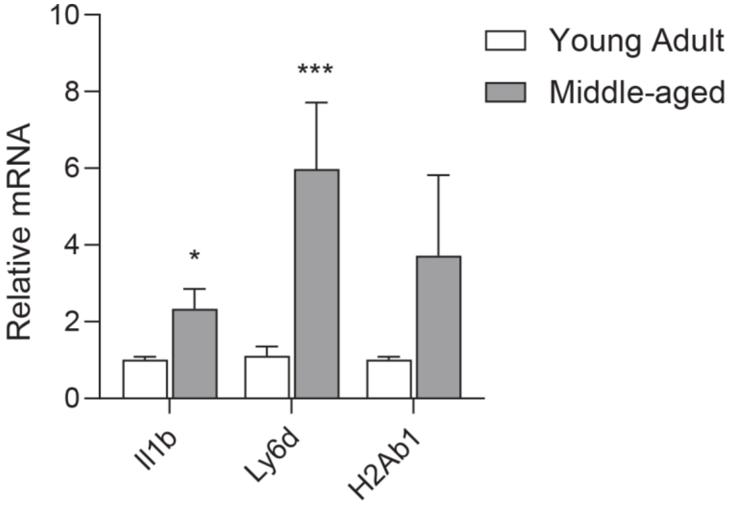

Supplementary Figure 2. Hypothalamic inflammatory gene expression in young and middle-aged mice. Inflammatory cytokine interleukin 1β (*Il1b*), lymphocyte antigen family 6 member D (*Ly6d*), and histocompatibility 2, class II antigen A, beta 1 (*H2Ab1*) gene expression. n = 6-7 per group. *p<0.05, ***p<0.001. Values are means ± SEM. Statistical analyses are shown in Supplementary File 1.

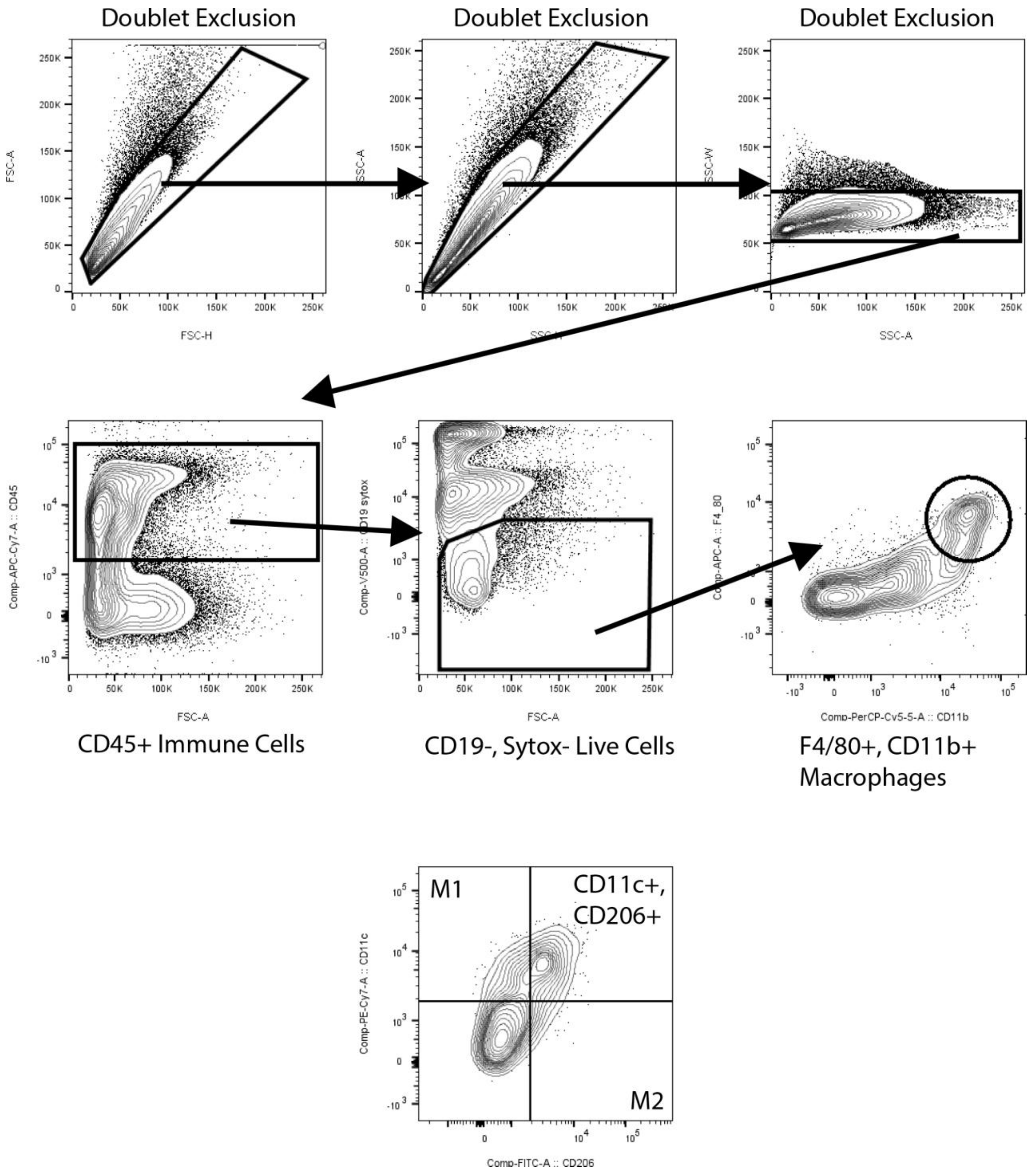

Supplementary Figure 3. Gonadal white adipose tissue stromal vascular fraction flow cytometry gating strategy.

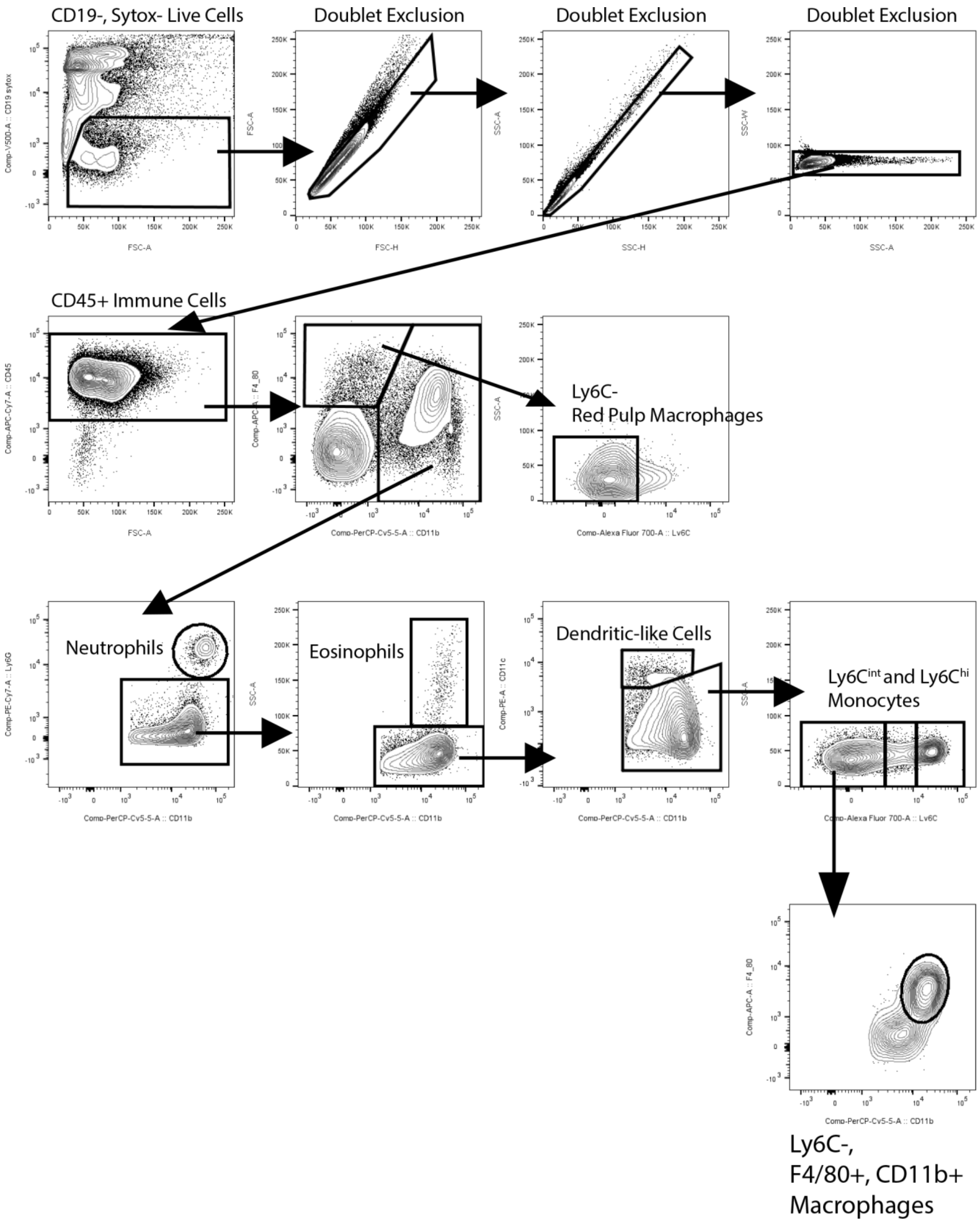

Supplementary Figure 4. Splenic flow cytometry gating strategy.

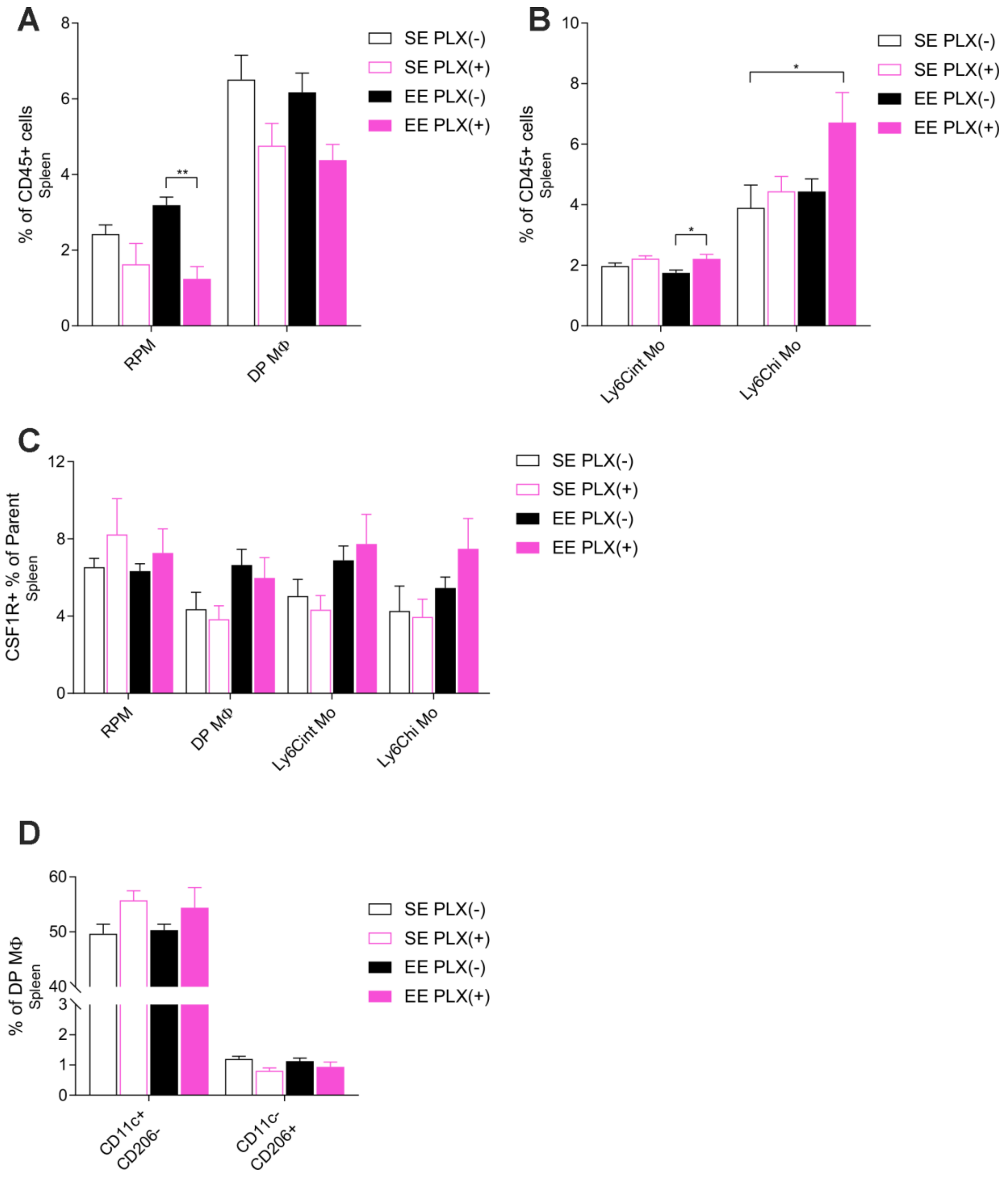

Supplementary Figure 5. Splenic monocyte and macrophage response to PLX5622 and environmental enrichment in middle-aged mice. (**A**) Splenic Ly6C- red pulp macrophages (RPM) and F4/80+, CD11b+ macrophages (DP MΦ). (**B**) Splenic Ly6C intermediate ($Ly6C^{int}$) and Ly6C high ($Ly6C^{hi}$) monocytes (Mo). (**C**) CSF1R+ percentage within each population. (**D**) DP MΦ polarization, M1: CD11c+, CD206-, M2: CD11c-, CD206+. (**A**–**D**) *n*=5-6 per group. **p*<0.05, ***p*<0.01. Values are means ± SEM. Statistical analyses are shown in Supplementary File 1.
