# Supplementary material for: CSF1R inhibitor PLX5622 and environmental enrichment additively improve metabolic outcomes in middle-aged female mice
Source: Aging (Albany NY). 2020 Feb 2;12(3):2101–22. doi: 10.18632/aging.102724 (PMC7041757; doi:10.18632/aging.102724)
Supplement: Supplementary Table 1 [file aging-12-102724-s001..pdf]

## SUPPLEMENTARY TABLE

**Supplementary Table 1. Antibodies used for flow cytometry.**

| Antibodies | Source | Catalog identifier |
|---|---|---|
| CD11c (Clone: HL3) | BD Biosciences | 553802 |
| Ly6C (Clone: AL21) | BD Biosciences | 561237 |
| CD45 (Clone: 30/F11) | BD Biosciences | 557659 |
| CD206 (Clone: C068C2) | BioLegend | 141704 |
| CD11b (Clone: M1/70) | BioLegend | 101228 |
| Ly6G (Clone: 1A8) | BioLegend | 127617 |
| CD11c (Clone: N418) | BioLegend | 117318 |
| CSF1R (Clone: AFS98) | BioLegend | 135513 |
| CD19 (Clone 6D5) | BioLegend | 115546 |
| F4/80 (Clone: BM8) | BioLegend | 123116 |
| Sytox Blue | Invitrogen | S34857 |
