# Supplementary material for: CSF1R inhibitor PLX5622 and environmental enrichment additively improve metabolic outcomes in middle-aged female mice
Source: Aging (Albany NY). 2020 Feb 2;12(3):2101–22. doi: 10.18632/aging.102724 (PMC7041757; doi:10.18632/aging.102724)
Supplement: Supplementary File 1 [file aging-12-102724-s003..pdf]

Figure 1a

Body Weight

Week 0

ANOVA table	SS	DF	MS	F (DFn, DFd)	P value	P value summary
Interaction	0.18225	1	0.18225	F (1, 36) = 0.0479953	P=0.827826	ns
PLX	0.04225	1	0.04225	F (1, 36) = 0.0111265	P=0.916579	ns
Housing	5.40225	1	5.40225	F (1, 36) = 1.42267	P=0.240766	ns
Residual	136.701	36	3.79725			

Holm-Sidak adjustment	Individual P Value	1-(1-α)^(1/i) comparison	j, asc. rank	Adjusted P Value	Summary
PLX(-):SE vs. PLX(-):EE	0.324788	0.010206218	1	0.859653767	ns
PLX(-):SE vs. PLX(+):SE	0.936424	0.05	5	0.936424	ns
PLX(-):SE vs. PLX(+):EE	0.447014	0.012741455	2	0.906490325	ns
PLX(-):EE vs. PLX(+):SE	0.364731				
PLX(-):EE vs. PLX(+):EE	0.81978	0.025320566	4	0.967520752	ns
PLX(+):SE vs. PLX(+):EE	0.495553	0.016952428	3	0.871634998	ns

Week 1

ANOVA table	SS	DF	MS	F (DFn, DFd)	P value	P value summary
Interaction	5.776	1	5.776	F (1, 36) = 1.39	P=0.246138	ns
PLX	33.489	1	33.489	F (1, 36) = 8.05917	P=0.007397	**
Housing	1.6	1	1.6	F (1, 36) = 0.385042	P=0.538826	ns
Residual	149.594	36	4.15539			

Holm-Sidak adjustment	Individual P Value	1-(1-α)^(1/i) comparison	j, asc. rank	Adjusted P Value	Summary
PLX(-):SE vs. PLX(-):EE	0.69525	0.05	5	0.69525	ns
PLX(-):SE vs. PLX(+):SE	0.248216	0.025320566	4	0.434820817	ns
PLX(-):SE vs. PLX(+):EE	0.019456	0.012741455	2	0.0755821	ns
PLX(-):EE vs. PLX(+):SE	0.125488				
PLX(-):EE vs. PLX(+):EE	0.007356	0.010206218	1	0.036242858	*
PLX(+):SE vs. PLX(+):EE	0.211374	0.016952428	3	0.509529069	ns

Week 2

ANOVA table	SS	DF	MS	F (DFn, DFd)	P value	P value summary
Interaction	3.481	1	3.481	F (1, 36) = 1.41786	P=0.241549	ns
PLX	27.889	1	27.889	F (1, 36) = 11.3596	P=0.001803	**
Housing	0.361	1	0.361	F (1, 36) = 0.14704	P=0.703635	ns
Residual	88.384	36	2.45511			

Holm-Sidak adjustment	Individual P Value	1-(1-α)^(1/i) comparison	j, asc. rank	Adjusted P Value	Summary
PLX(-):SE vs. PLX(-):EE	0.273032	0.025320566	4	0.471517527	ns
PLX(-):SE vs. PLX(+):SE	0.132001	0.016952428	3	0.346030228	ns
PLX(-):SE vs. PLX(+):EE	0.041684	0.012741455	2	0.156597359	ns
PLX(-):EE vs. PLX(+):SE	0.011756				
PLX(-):EE vs. PLX(+):EE	0.002679	0.010206218	1	0.013323422	*
PLX(+):SE vs. PLX(+):EE	0.571659	0.05	5	0.571659	ns

Week 3

ANOVA table	SS	DF	MS	F (DFn, DFd)	P value	P value summary
Interaction	0.625	1	0.625	F (1, 36) = 0.242285	P=0.625549	ns
PLX	8.1	1	8.1	F (1, 36) = 3.14001	P=0.084858	ns
Housing	1.444	1	1.444	F (1, 36) = 0.559774	P=0.459211	ns
Residual	92.866	36	2.57961			

Holm-Sidak adjustment	Individual P Value	1-(1-α)^(1/i) comparison	j, asc. rank	Adjusted P Value	Summary
PLX(-):SE vs. PLX(-):EE	0.857392	0.05	5	0.857392	ns
PLX(-):SE vs. PLX(+):SE	0.371512	0.016952428	3	0.751749021	ns
PLX(-):SE vs. PLX(+):EE	0.083179	0.010206218	1	0.35222712	ns
PLX(-):EE vs. PLX(+):SE	0.473772				
PLX(-):EE vs. PLX(+):EE	0.118105	0.012741455	2	0.395122373	ns
PLX(+):SE vs. PLX(+):EE	0.386249	0.025320566	4	0.62330971	ns

Week 4

ANOVA table	SS	DF	MS	F (DFn, DFd)	P value	P value summary
Interaction	0.15625	1	0.15625	F (1, 36) = 0.055544	P=0.815018	ns
PLX	6.80625	1	6.80625	F (1, 36) = 2.4195	P=0.128582	ns
Housing	2.45025	1	2.45025	F (1, 36) = 0.871019	P=0.356891	ns
Residual	101.271	36	2.81308			

Holm-Sidak adjustment	Individual P Value	1-(1-α)^(1/i) comparison	j, asc. rank	Adjusted P Value	Summary
PLX(-):SE vs. PLX(-):EE	0.624809	0.05	5	0.624809	ns
PLX(-):SE vs. PLX(+):SE	0.356915	0.016952428	3	0.73404685	ns
PLX(-):SE vs. PLX(+):EE	0.086935	0.010206218	1	0.365387711	ns
PLX(-):EE vs. PLX(+):SE	0.6626				
PLX(-):EE vs. PLX(+):EE	0.213455	0.012741455	2	0.61726842	ns
PLX(+):SE vs. PLX(+):EE	0.41392	0.025320566	4	0.656510234	ns

Week 5

ANOVA table	SS	DF	MS	F (DFn, DFd)	P value	P value summary
Interaction	0.81225	1	0.81225	F (1, 36) = 0.202096	P=0.65573	ns
PLX	12.8823	1	12.8823	F (1, 36) = 3.20523	P=0.081817	ns
Housing	2.75625	1	2.75625	F (1, 36) = 0.685781	P=0.41306	ns
Residual	144.689	36	4.01914			

Holm-Sidak adjustment	Individual P Value	1-(1-α)^(1/i) comparison	j, asc. rank	Adjusted P Value	Summary
PLX(-):SE vs. PLX(-):EE	0.790467	0.05	5	0.790467	ns
PLX(-):SE vs. PLX(+):SE	0.349418	0.016952428	3	0.724636654	ns
PLX(-):SE vs. PLX(+):EE	0.072312	0.010206218	1	0.312916223	ns
PLX(-):EE vs. PLX(+):SE	0.50062				
PLX(-):EE vs. PLX(+):EE	0.12198	0.012741455	2	0.40568371	ns
PLX(+):SE vs. PLX(+):EE	0.372294	0.025320566	4	0.605985178	ns

Figure 1b

Body Weight, % Change

Week 1

ANOVA table	SS	DF	MS	F (DFn, DFd)	P value	P value summary
Interaction	58.7893	1	58.7893	F (1, 36) = 2.65333	P=0.112055	ns
PLX	484.938	1	484.938	F (1, 36) = 21.8866	P=0.00004	****
Housing	166.672	1	166.672	F (1, 36) = 7.52238	P=0.009435	**
Residual	797.646	36	22.1568			

Holm-Sidak adjustment	Individual P Value	1-(1-α)^(1/i) comparison	j, asc. rank	Adjusted P Value	Summary
PLX(-):SE vs. PLX(-):EE	0.436103	0.05	5	0.436103	ns
PLX(-):SE vs. PLX(+):SE	0.03782	0.025320566	4	0.074209648	ns
PLX(-):SE vs. PLX(+):EE	0.000007	0.010206218	1	3.49995E-05	****
PLX(-):EE vs. PLX(+):SE	0.179577				
PLX(-):EE vs. PLX(+):EE	0.000077	0.012741455	2	0.000307964	***
PLX(+):SE vs. PLX(+):EE	0.003835	0.016952428	3	0.011460935	*

Week 2

ANOVA table	SS	DF	MS	F (DFn, DFd)	P value	P value summary
Interaction	31.4495	1	31.4495	F (1, 36) = 1.11104	P=0.29888	ns
PLX	415.728	1	415.728	F (1, 36) = 14.6867	P=0.00049	***
Housing	36.7911	1	36.7911	F (1, 36) = 1.29975	P=0.261788	ns
Residual	1019.03	36	28.3064			

Holm-Sidak adjustment	Individual P Value	1-(1-α)^(1/i) comparison	j, asc. rank	Adjusted P Value	Summary
PLX(-):SE vs. PLX(-):EE	0.951843	0.05	5	0.951843	ns
PLX(-):SE vs. PLX(+):SE	0.057224	0.016952428	3	0.162035626	ns
PLX(-):SE vs. PLX(+):EE	0.001204	0.010206218	1	0.006005521	**
PLX(-):EE vs. PLX(+):SE	0.064965				
PLX(-):EE vs. PLX(+):EE	0.001426	0.012741455	2	0.005691811	**
PLX(+):SE vs. PLX(+):EE	0.129535	0.025320566	4	0.242290684	ns

Week 3

ANOVA table	SS	DF	MS	F (DFn, DFd)	P value	P value summary
Interaction	1.93248	1	1.93248	F (1, 36) = 0.084173	P=0.773385	ns
PLX	115.158	1	115.158	F (1, 36) = 5.01595	P=0.031384	*
Housing	177.974	1	177.974	F (1, 36) = 7.75201	P=0.008498	**
Residual	826.505	36	22.9585			

Holm-Sidak adjustment	Individual P Value	1-(1-α)^(1/i) comparison	j, asc. rank	Adjusted P Value	Summary
PLX(-):SE vs. PLX(-):EE	0.086284	0.025320566	4	0.165123071	ns
PLX(-):SE vs. PLX(+):SE	0.176553	0.05	5	0.176553	ns
PLX(-):SE vs. PLX(+):EE	0.001087	0.010206218	1	0.005423197	**
PLX(-):EE vs. PLX(+):SE	0.70243				
PLX(-):EE vs. PLX(+):EE	0.082063	0.016952428	3	0.226538632	ns
PLX(+):SE vs. PLX(+):EE	0.036367	0.012741455	2	0.137723289	ns

Week 4

ANOVA table	SS	DF	MS	F (DFn, DFd)	P value	P value summary
Interaction	0.0141	1	0.0141	F (1, 36) = 0.000583183	P=0.980867	ns
PLX	97.0042	1	97.0042	F (1, 36) = 4.01214	P=0.052747	ns
Housing	219.01	1	219.01	F (1, 36) = 9.05835	P=0.004755	**
Residual	870.397	36	24.1777			

Holm-Sidak adjustment	Individual P Value	1-(1-α)^(1/i) comparison	j, asc. rank	Adjusted P Value	Summary
PLX(-):SE vs. PLX(-):EE	0.038751	0.012741455	2	0.146224665	ns
PLX(-):SE vs. PLX(+):SE	0.160362	0.025320566	4	0.295008029	ns
PLX(-):SE vs. PLX(+):EE	0.001111	0.010206218	1	0.00554267	**
PLX(-):EE vs. PLX(+):SE	0.481159				
PLX(-):EE vs. PLX(+):EE	0.170286	0.05	5	0.170286	ns
PLX(+):SE vs. PLX(+):EE	0.041773	0.016952428	3	0.120156943	ns

Week 5

ANOVA table	SS	DF	MS	F (DFn, DFd)	P value	P value summary
Interaction	2.90952	1	2.90952	F (1, 36) = 0.121288	P=0.72967	ns
PLX	178.616	1	178.616	F (1, 36) = 7.4459	P=0.009772	**
Housing	237.247	1	237.247	F (1, 36) = 9.89001	P=0.003325	**
Residual	863.587	36	23.9885			

Holm-Sidak adjustment	Individual P Value	1-(1-α)^(1/i) comparison	j, asc. rank	Adjusted P Value	Summary
PLX(-):SE vs. PLX(-):EE	0.055683	0.025320566	4	0.108265404	ns
PLX(-):SE vs. PLX(+):SE	0.100984	0.05	5	0.100984	ns
PLX(-):SE vs. PLX(+):EE	0.000192	0.010206218	1	0.000959631	***
PLX(-):EE vs. PLX(+):SE	0.770262				
PLX(-):EE vs. PLX(+):EE	0.036218	0.016952428	3	0.104766278	ns
PLX(+):SE vs. PLX(+):EE	0.018385	0.012741455	2	0.071536694	ns

Figure 1c

Relative Food Intake

ANOVA table	SS	DF	MS	F (DFn, DFd)	P value	P value summary
Interaction	0.000124664	1	0.000124664	F (1, 44) = 0.507313	P=0.480064	ns
PLX	4.34181E-06	1	4.34181E-06	F (1, 44) = 0.0176687	P=0.89486	ns
Housing	0.00722248	1	0.00722248	F (1, 44) = 29.3914	P=0.000002	****
Residual	0.0108123	44	0.000245735			

Holm-Sidak adjustment	Individual P Value	1-(1-α)^(1/i) comparison	j, asc. rank	Adjusted P Value	Summary
PLX(-):SE vs. PLX(-):EE	0.001765	0.016952428	3	0.00528566	**
PLX(-):SE vs. PLX(+):SE	0.684049	0.05	5	0.684049	ns
PLX(-):SE vs. PLX(+):EE	0.000299	0.012741455	2	0.001195464	**
PLX(-):EE vs. PLX(+):SE	0.000529				
PLX(-):EE vs. PLX(+):EE	0.553147	0.025320566	4	0.800322396	ns
PLX(+):SE vs. PLX(+):EE	0.000083	0.010206218	1	0.000414931	***

Figure 1d

Body Fat %

ANOVA table	SS	DF	MS	F (DFn, DFd)	P value	P value summary
Interaction	36.6588	1	36.6588	F (1, 36) = 2.2754	P=0.140168	ns
PLX	121.7	1	121.7	F (1, 36) = 7.55385	P=0.009301	**
Housing	442.604	1	442.604	F (1, 36) = 27.4722	P=0.000007	****
Residual	579.995	36	16.111			

Holm-Sidak adjustment	Individual P Value	1-(1-α)^(1/i) comparison	j, asc. rank	Adjusted P Value	Summary
PLX(-):SE vs. PLX(-):EE	0.012192	0.025320566	4	0.024235355	*
PLX(-):SE vs. PLX(+):SE	0.386408	0.05	5	0.386408	ns
PLX(-):SE vs. PLX(+):EE	0.000002	0.010206218	1	9.99996E-06	****
PLX(-):EE vs. PLX(+):SE	0.086423				
PLX(-):EE vs. PLX(+):EE	0.00475	0.016952428	3	0.01418242	*
PLX(+):SE vs. PLX(+):EE	0.00003	0.012741455	2	0.000119995	***

Figure 1e

Lean Weight %

ANOVA table	SS	DF	MS	F (DFn, DFd)	P value	P value summary
Interaction	9.20065	1	9.20065	F (1, 36) = 0.674162	P=0.417009	ns
PLX	169.769	1	169.769	F (1, 36) = 12.4395	P=0.001168	**
Housing	456.638	1	456.638	F (1, 36) = 33.4594	P=0.000001	****
Residual	491.311	36	13.6475			

Holm-Sidak adjustment	Individual P Value	1-(1-α)^(1/i) comparison	j, asc. rank	Adjusted P Value	Summary
PLX(-):SE vs. PLX(-):EE	0.001226	0.016952428	3	0.003673493	**
PLX(-):SE vs. PLX(+):SE	0.063681	0.05	5	0.063681	ns
PLX(-):SE vs. PLX(+):EE	<0.000001	0.010206218	1	4.99999E-06	****
PLX(-):EE vs. PLX(+):SE	0.119175				
PLX(-):EE vs. PLX(+):EE	0.004008	0.025320566	4	0.007999936	**
PLX(+):SE vs. PLX(+):EE	0.000041	0.012741455	2	0.00016399	***

Figure 1g

Glucose Tolerance Test AUC

ANOVA table	SS	DF	MS	F (DFn, DFd)	P value	P value summary
Interaction	1076660	1	1076660	F (1, 36) = 0.0279455	P=0.868172	ns
PLX	98588150	1	98588150	F (1, 36) = 2.55893	P=0.118414	ns
Housing	356185160	1	356185160	F (1, 36) = 9.24505	P=0.004384	**
Residual	1386975786	36	38527105			

Holm-Sidak adjustment	Individual P Value	1-(1-α)^(1/i) comparison	j, asc. rank	Adjusted P Value	Summary
PLX (-):SE vs. PLX (-):EE	0.029414	0.012741455	2	0.112565945	ns
PLX (-):SE vs. PLX (+):SE	0.219603	0.025320566	4	0.390980522	ns
PLX (-):SE vs. PLX (+):EE	0.002302	0.010206218	1	0.01145713	*
PLX (-):EE vs. PLX (+):SE	0.315062				
PLX (-):EE vs. PLX (+):EE	0.317854	0.05	5	0.317854	ns
PLX (+):SE vs. PLX (+):EE	0.049605	0.016952428	3	0.141555093	ns

Figure 1h

Fasting Blood Glucose

ANOVA table	SS	DF	MS	F (DFn, DFd)	P value	P value summary
Interaction	148.225	1	148.225	F (1, 36) = 0.219152	P=0.642509	ns
PLX	11055.6	1	11055.6	F (1, 36) = 16.3458	P=0.000266	***
Housing	3441.03	1	3441.03	F (1, 36) = 5.08758	P=0.030271	*
Residual	24348.9	36	676.358			

Holm-Sidak adjustment	Individual P Value	α/i comparison	j, asc. rank	Adjusted P Value	Summary
PLX (-):SE vs. PLX (-):EE	0.214388	0.05	5	0.214388	ns
PLX (-):SE vs. PLX (+):SE	0.016008	0.016666667	3	0.047259334	*
PLX (-):SE vs. PLX (+):EE	0.000079	0.01	1	0.000394938	***
PLX (-):EE vs. PLX (+):SE	0.214388				
PLX (-):EE vs. PLX (+):EE	0.002947	0.0125	2	0.011735993	*
PLX (+):SE vs. PLX (+):EE	0.062037	0.025	4	0.120225411	ns

Figure 1j

Pyruvate Tolerance Test AUC

ANOVA table	SS	DF	MS	F (DFn, DFd)	P value	P value summary
Interaction	2134440	1	2134440	F (1, 36) = 0.440869	P=0.510934	ns
PLX	22119126	1	22119126	F (1, 36) = 4.56871	P=0.039424	*
Housing	60663690	1	60663690	F (1, 36) = 12.5301	P=0.001126	**
Residual	174291806	36	4841439			

Holm-Sidak adjustment	Individual P Value	1-(1-α)^(1/i) comparison	j, asc. rank	Adjusted P Value	Summary
PLX (-):SE vs. PLX (-):EE	0.00524	0.012741455	2	0.020795829	*
PLX (-):SE vs. PLX (+):SE	0.05528	0.025320566	4	0.107504122	ns
PLX (-):SE vs. PLX (+):EE	0.000289	0.010206218	1	0.001444165	**
PLX (-):EE vs. PLX (+):SE	0.328011				
PLX (-):EE vs. PLX (+):EE	0.304403	0.05	5	0.304403	ns
PLX (+):SE vs. PLX (+):EE	0.049424	0.016952428	3	0.141064534	ns

Figure 2a

Relative Tissue Weights at Sacrifice

BAT

ANOVA table	SS	DF	MS	F (DFn, DFd)	P value	P value summary
Interaction	0.000247042	1	0.000247042	F (1, 20) = 0.0904459	P=0.766715	ns
PLX	0.00585937	1	0.00585937	F (1, 20) = 2.14521	P=0.158559	ns
Housing	0.00885504	1	0.00885504	F (1, 20) = 3.24197	P=0.086876	ns
Residual	0.0546275	20	0.00273138			

Holm-Sidak adjustment	Individual P Value	1-(1-α)^(1/i) comparison	j, asc. rank	Adjusted P Value	Summary
PLX(-):SE vs. PLX(-):EE	0.301547	0.025320566	4	0.512163407	ns
PLX(-):SE vs. PLX(+):SE	0.420208	0.05	5	0.420208	ns
PLX(-):SE vs. PLX(+):EE	0.031759	0.010206218	1	0.149023937	ns
PLX(-):EE vs. PLX(+):SE	0.814675				
PLX(-):EE vs. PLX(+):EE	0.226329	0.016952428	3	0.536906213	ns
PLX(+):SE vs. PLX(+):EE	0.152916	0.012741455	2	0.485120127	ns

iWAT

ANOVA table	SS	DF	MS	F (DFn, DFd)	P value	P value summary
Interaction	0.0247042	1	0.0247042	F (1, 20) = 0.345517	P=0.563241	ns
PLX	1.5965	1	1.5965	F (1, 20) = 22.329	P=0.00013	***
Housing	0.802273	1	0.802273	F (1, 20) = 11.2207	P=0.00319	**
Residual	1.42998	20	0.0714991			

Holm-Sidak adjustment	Individual P Value	1-(1-α)^(1/i) comparison	j, asc. rank	Adjusted P Value	Summary
PLX(-):SE vs. PLX(-):EE	0.064958	0.05	5	0.064958	ns
PLX(-):SE vs. PLX(+):SE	0.00836	0.016952428	3	0.024870915	*
PLX(-):SE vs. PLX(+):EE	0.000014	0.010206218	1	6.9998E-05	****
PLX(-):EE vs. PLX(+):SE	0.342315				
PLX(-):EE vs. PLX(+):EE	0.001241	0.012741455	2	0.004954767	**
PLX(+):SE vs. PLX(+):EE	0.011448	0.025320566	4	0.022764943	*

gWAT

ANOVA table	SS	DF	MS	F (DFn, DFd)	P value	P value summary
Interaction	0.182115	1	0.182115	F (1, 36) = 0.547128	P=0.46429	ns
PLX	4.88531	1	4.88531	F (1, 36) = 14.6769	P=0.000492	***
Housing	6.70515	1	6.70515	F (1, 36) = 20.1443	P=0.000071	****
Residual	11.9828	36	0.332856			

Holm-Sidak adjustment	Individual P Value	1-(1-α)^(1/i) comparison	j, asc. rank	Adjusted P Value	Summary
PLX(-):SE vs. PLX(-):EE	0.011865	0.05	5	0.035406	*
PLX(-):SE vs. PLX(+):SE	0.035406	0.025320566	4	0.023589222	*
PLX(-):SE vs. PLX(+):EE	<0.000001	0.010206218	1	4.99999E-06	****
PLX(-):EE vs. PLX(+):SE	0.644942				
PLX(-):EE vs. PLX(+):EE	0.00263	0.012741455	2	0.002888865	**
PLX(+):SE vs. PLX(+):EE	0.000723	0.016952428	3	0.007869267	**

rWAT

ANOVA table	SS	DF	MS	F (DFn, DFd)	P value	P value summary
Interaction	0.000222042	1	0.000222042	F (1, 20) = 0.0255449	P=0.87462	ns
PLX	0.29062	1	0.29062	F (1, 20) = 33.4345	P=0.000012	****
Housing	0.088695	1	0.088695	F (1, 20) = 10.204	P=0.004554	**
Residual	0.173844	20	0.00869222			

Holm-Sidak adjustment	Individual P Value	1-(1-α)^(1/i) comparison	j, asc. rank	Adjusted P Value	Summary
PLX(-):SE vs. PLX(-):EE	0.044349	0.05	5	0.044349	*
PLX(-):SE vs. PLX(+):SE	0.000745	0.016952428	3	0.002233335	**
PLX(-):SE vs. PLX(+):EE	0.000003	0.010206218	1	1.49999E-05	****
PLX(-):EE vs. PLX(+):SE	0.082209				
PLX(-):EE vs. PLX(+):EE	0.000439	0.012741455	2	0.001754844	**
PLX(+):SE vs. PLX(+):EE	0.027853	0.025320566	4	0.05493021	ns

Liver

ANOVA table	SS	DF	MS	F (DFn, DFd)	P value	P value summary
Interaction	0.489918	1	0.489918	F (1, 20) = 1.13053	P=0.300333	ns
PLX	0.519498	1	0.519498	F (1, 20) = 1.19878	P=0.286576	ns
Housing	0.47012	1	0.47012	F (1, 20) = 1.08484	P=0.310045	ns
Residual	8.66709	20	0.433355			

Holm-Sidak adjustment	Individual P Value	1-(1-α)^(1/i) comparison	j, asc. rank	Adjusted P Value	Summary
PLX(-):SE vs. PLX(-):EE	0.152263	0.012741455	2	0.483530649	ns
PLX(-):SE vs. PLX(+):SE	0.982379	0.025320566	4	0.9996895	ns
PLX(-):SE vs. PLX(+):EE	0.970291	0.016952428	3	0.999973778	ns
PLX(-):EE vs. PLX(+):SE	0.146505				
PLX(-):EE vs. PLX(+):EE	0.142659	0.010206218	1	0.536800624	ns
PLX(+):SE vs. PLX(+):EE	0.987907	0.05	5	0.987907	ns

Soleus

ANOVA table	SS	DF	MS	F (DFn, DFd)	P value	P value summary
Interaction	1.06667E-05	1	1.06667E-05	F (1, 20) = 0.234175	P=0.633702	ns
PLX	0.0005415	1	0.0005415	F (1, 20) = 11.888	P=0.002544	**
Housing	0.000912667	1	0.000912667	F (1, 20) = 20.0366	P=0.000231	***
Residual	0.000911	20	0.00004555			

Holm-Sidak adjustment	Individual P Value	1-(1-α)^(1/i) comparison	j, asc. rank	Adjusted P Value	Summary
PLX(-):SE vs. PLX(-):EE	0.010508	0.016952428	3	0.031193906	*
PLX(-):SE vs. PLX(+):SE	0.049022	0.05	5	0.049022	*
PLX(-):SE vs. PLX(+):EE	0.000017	0.010206218	1	8.49971E-05	****
PLX(-):EE vs. PLX(+):SE	0.47557				
PLX(-):EE vs. PLX(+):EE	0.011551	0.025320566	4	0.022968574	*
PLX(+):SE vs. PLX(+):EE	0.002217	0.012741455	2	0.008838553	**

Gastrocnemius

ANOVA table	SS	DF	MS	F (DFn, DFd)	P value	P value summary
Interaction	0.029051	1	0.029051	F (1, 20) = 6.25948	P=0.021148	*
PLX	0.028085	1	0.028085	F (1, 20) = 6.05134	P=0.023124	*
Housing	0.00495938	1	0.00495938	F (1, 20) = 1.06857	P=0.313607	ns
Residual	0.0928225	20	0.00464113			

Holm-Sidak adjustment	Individual P Value	1-(1-α)^(1/i) comparison	j, asc. rank	Adjusted P Value	Summary
PLX(-):SE vs. PLX(-):EE	0.311585	0.025320566	4	0.526084788	ns
PLX(-):SE vs. PLX(+):SE	0.976631	0.05	5	0.976631	ns
PLX(-):SE vs. PLX(+):EE	0.022616	0.016952428	3	0.066325117	ns
PLX(-):EE vs. PLX(+):SE	0.325262				
PLX(-):EE vs. PLX(+):EE	0.002211	0.010206218	1	0.011006223	*
PLX(+):SE vs. PLX(+):EE	0.021231	0.012741455	2	0.082257545	ns

Spleen

ANOVA table	SS	DF	MS	F (DFn, DFd)	P value	P value summary
Interaction	0.0400167	1	0.0400167	F (1, 20) = 0.245614	P=0.625582	ns
PLX	0.949628	1	0.949628	F (1, 20) = 5.82862	P=0.025472	*
Housing	0.38964	1	0.38964	F (1, 20) = 2.39153	P=0.137672	ns
Residual	3.2585	20	0.162925			

Holm-Sidak adjustment	Individual P Value	1-(1-α)^(1/i) comparison	j, asc. rank	Adjusted P Value	Summary
PLX(-):SE vs. PLX(-):EE	0.164237	0.012741455	2	0.51209804	ns
PLX(-):SE vs. PLX(+):SE	0.189995	0.016952428	3	0.468549158	ns
PLX(-):SE vs. PLX(+):EE	0.54638	0.05	5	0.54638	ns
PLX(-):EE vs. PLX(+):SE	0.011041				
PLX(-):EE vs. PLX(+):EE	0.052905	0.010206218	1	0.237977633	ns
PLX(+):SE vs. PLX(+):EE	0.466077	0.025320566	4	0.71492623	ns

Figure 2b

Serum Leptin

ANOVA table	SS	DF	MS	F (DFn, DFd)	P value	P value summary
Interaction	27562.9	1	27562.9	F (1, 20) = 0.00509256	P=0.943818	ns
PLX	27168398	1	27168398	F (1, 20) = 5.01967	P=0.036574	*
Housing	47755901	1	47755901	F (1, 20) = 8.82344	P=0.007562	**
Residual	108247836	20	5412392			

Holm-Sidak adjustment	Individual P Value	1-(1-α)^(1/i) comparison	j, asc. rank	Adjusted P Value	Summary
PLX(-):SE vs. PLX(-):EE	0.05371	0.016952428	3	0.152630648	ns
PLX(-):SE vs. PLX(+):SE	0.14075	0.05	5	0.14075	ns
PLX(-):SE vs. PLX(+):EE	0.001469	0.010206218	1	0.007323452	**
PLX(-):EE vs. PLX(+):SE	0.6114				
PLX(-):EE vs. PLX(+):EE	0.117753	0.025320566	4	0.221640231	ns
PLX(+):SE vs. PLX(+):EE	0.043892	0.012741455	2	0.164343476	ns

Figure 3e

Microglia Cell Count

ANOVA table	SS	DF	MS	F (DFn, DFd)	P value	P value summary
Interaction	4.87674	1	4.87674	F (1, 12) = 0.88264	P=0.366006	ns
PLX	955.84	1	955.84	F (1, 12) = 172.997	P<0.000001	****
Housing	9.50696	1	9.50696	F (1, 12) = 1.72066	P=0.214149	ns
Residual	66.3021	12	5.52517			

Holm-Sidak adjustment	Individual P Value	1-(1-α)^(1/i) comparison	j, asc. rank	Adjusted P Value	Summary
PLX(-):SE vs. PLX(-):EE	0.137401	0.025320566	4	0.255922965	ns
PLX(-):SE vs. PLX(+):SE	0.000002	0.014548362	2.5	6.99998E-06	****
PLX(-):SE vs. PLX(+):EE	0.000002	0.014548362	2.5	6.99998E-06	****
PLX(-):EE vs. PLX(+):SE	<0.000001				
PLX(-):EE vs. PLX(+):EE	<0.000001	0.010206218	1	4.99999E-06	****
PLX(+):SE vs. PLX(+):EE	0.796845	0.05	5	0.796845	ns

Figure 4a

Hypothalamic qRT-PCR

Bdnf

ANOVA table	SS	DF	MS	F (DFn, DFd)	P value	P value summary
Interaction	0.00532091	1	0.00532091	F (1, 20) = 0.0206036	P=0.887301	ns
PLX	0.00479685	1	0.00479685	F (1, 20) = 0.0185743	P=0.892956	ns
Housing	6.88655	1	6.88655	F (1, 20) = 26.666	P=0.000047	****
Residual	5.16504	20	0.258252			

Holm-Sidak adjustment	Individual P Value	1-(1-α)^(1/i) comparison	j, asc. rank	Adjusted P Value	Summary
PLX(-):SE vs. PLX(-):EE	0.002009	0.016952428	3	0.0060149	**
PLX(-):SE vs. PLX(+):SE	0.845146	0.025320566	4	0.976020239	ns
PLX(-):SE vs. PLX(+):EE	0.001985	0.012741455	2	0.00791639	**
PLX(-):EE vs. PLX(+):SE	0.001268				
PLX(-):EE vs. PLX(+):EE	0.995959	0.05	5	0.995959	ns
PLX(+):SE vs. PLX(+):EE	0.001253	0.010206218	1	0.00624932	**

Npy

ANOVA table	SS	DF	MS	F (DFn, DFd)	P value	P value summary
Interaction	0.00144201	1	0.00144201	F (1, 20) = 0.00879223	P=0.926227	ns
PLX	0.997188	1	0.997188	F (1, 20) = 6.08005	P=0.022839	*
Housing	41.3503	1	41.3503	F (1, 20) = 252.121	P<0.000001	****
Residual	3.2802	20	0.16401			

Holm-Sidak adjustment	Individual P Value	1-(1-α)^(1/i) comparison	j, asc. rank	Adjusted P Value	Summary
PLX(-):SE vs. PLX(-):EE	<0.000001	0.012741455	2	3.99999E-06	****
PLX(-):SE vs. PLX(+):SE	0.109052	0.05	5	0.109052	ns
PLX(-):SE vs. PLX(+):EE	<0.000001	0.012741455	2	3.99999E-06	****
PLX(-):EE vs. PLX(+):SE	<0.000001				
PLX(-):EE vs. PLX(+):EE	0.08537	0.025320566	4	0.163451963	ns
PLX(+):SE vs. PLX(+):EE	<0.000001	0.012741455	2	3.99999E-06	****

Pomc

ANOVA table	SS	DF	MS	F (DFn, DFd)	P value	P value summary
Interaction	0.436258	1	0.436258	F (1, 20) = 2.06713	P=0.165968	ns
PLX	0.303989	1	0.303989	F (1, 20) = 1.4404	P=0.244099	ns
Housing	25.5176	1	25.5176	F (1, 20) = 120.911	P<0.000001	****
Residual	4.22091	20	0.211045			

Holm-Sidak adjustment	Individual P Value	1-(1-α)^(1/i) comparison	j, asc. rank	Adjusted P Value	Summary
PLX(-):SE vs. PLX(-):EE	<0.000001	0.012741455	2	3.99999E-06	****
PLX(-):SE vs. PLX(+):SE	0.868272	0.05	5	0.868272	ns
PLX(-):SE vs. PLX(+):EE	0.000001	0.012741455	2	3.99999E-06	****
PLX(-):EE vs. PLX(+):SE	<0.000001				
PLX(-):EE vs. PLX(+):EE	0.076881	0.025320566	4	0.147851312	ns
PLX(+):SE vs. PLX(+):EE	0.000001	0.012741455	2	3.99999E-06	****

Crh

ANOVA table	SS	DF	MS	F (DFn, DFd)	P value
Interaction	5.46046	1	5.46046	F (1, 20) = 2.66895	P=0.117967
PLX	8.77288	1	8.77288	F (1, 20) = 4.28799	P=0.051539
Housing	0.844303	1	0.844303	F (1, 20) = 0.412677	P=0.52791
Residual	40.9184	20	2.04592		

Holm-Sidak adjustment	Individual P Value	1-(1-α)^(1/i) comparison	j, asc. rank	Adjusted P Value	Summary
PLX(-):SE vs. PLX(-):EE	0.123192	0.016952428	3	0.325916788	ns
PLX(-):SE vs. PLX(+):SE	0.016421	0.010206218	1	0.079452424	ns
PLX(-):SE vs. PLX(+):EE	0.069438	0.012741455	2	0.250138156	ns
PLX(-):EE vs. PLX(+):SE	0.324562				
PLX(-):EE vs. PLX(+):EE	0.760485	0.05	5	0.760485	ns
PLX(+):SE vs. PLX(+):EE	0.49141	0.025320566	4	0.741336212	ns

Gnrh

ANOVA table	SS	DF	MS	F (DFn, DFd)	P value	P value summary
Interaction	0.000635747	1	0.000635747	F (1, 20) = 0.00141985	P=0.970316	ns
PLX	0.27774	1	0.27774	F (1, 20) = 0.62029	P=0.440174	ns
Housing	0.977853	1	0.977853	F (1, 20) = 2.18389	P=0.155038	ns
Residual	8.95515	20	0.447758			

Holm-Sidak adjustment	Individual P Value	1-(1-α)^(1/i) comparison	j, asc. rank	Adjusted P Value	Summary
PLX(-):SE vs. PLX(-):EE	0.296659	0.012741455	2	0.755283226	ns
PLX(-):SE vs. PLX(+):SE	0.566046	0.025320566	4	0.811683926	ns
PLX(-):SE vs. PLX(+):EE	0.124862	0.010206218	1	0.486686472	ns
PLX(-):EE vs. PLX(+):SE	0.630819				
PLX(-):EE vs. PLX(+):EE	0.601769	0.05	5	0.601769	ns
PLX(+):SE vs. PLX(+):EE	0.320688	0.016952428	3	0.686521428	ns

Gfap

ANOVA table	SS	DF	MS	F (DFn, DFd)	P value	P value summary
Interaction	0.0460589	1	0.0460589	F (1, 20) = 0.405233	P=0.531618	ns
PLX	0.164911	1	0.164911	F (1, 20) = 1.45091	P=0.242443	ns
Housing	0.00231224	1	0.00231224	F (1, 20) = 0.0203434	P=0.888009	ns
Residual	2.27321	20	0.113661			

Holm-Sidak adjustment	Individual P Value	1-(1-α)^(1/i) comparison	j, asc. rank	Adjusted P Value	Summary
PLX(-):SE vs. PLX(-):EE	0.587748	0.016952428	3	0.929937067	ns
PLX(-):SE vs. PLX(+):SE	0.207758	0.010206218	1	0.687903203	ns
PLX(-):SE vs. PLX(+):EE	0.352169	0.012741455	2	0.823864497	ns
PLX(-):EE vs. PLX(+):SE	0.461468				
PLX(-):EE vs. PLX(+):EE	0.692232	0.025320566	4	0.905278858	ns
PLX(+):SE vs. PLX(+):EE	0.730534	0.05	5	0.730534	ns

Figure 4b

Hypothalamic qRT-PCR

Tmem119

ANOVA table	SS	DF	MS	F (DFn, DFd)	P value	P value summary
Interaction	2.26982E-05	1	2.26982E-05	F (1, 20) = 0.000104984	P=0.991926	ns
PLX	35.5233	1	35.5233	F (1, 20) = 164.304	P<0.000001	****
Housing	17.3723	1	17.3723	F (1, 20) = 80.351	P<0.000001	****
Residual	4.3241	20	0.216205			

Holm-Sidak adjustment	Individual P Value	1-(1-α)^(1/i) comparison	j, asc. rank	Adjusted P Value	Summary
PLX(-):SE vs. PLX(-):EE	0.000004	0.025320566	4	7.99998E-06	****
PLX(-):SE vs. PLX(+):SE	<0.000001	0.011333793	1.5	4.49999E-06	****
PLX(-):SE vs. PLX(+):EE	0.013033	0.05	5	0.013033	*
PLX(-):EE vs. PLX(+):SE	<0.000001				
PLX(-):EE vs. PLX(+):EE	<0.000001	0.011333793	1.5	4.49999E-06	****
PLX(+):SE vs. PLX(+):EE	0.000003	0.016952428	3	8.99997E-06	****

H2Ab1

ANOVA table	SS	DF	MS	F (DFn, DFd)	P value	P value summary
Interaction	0.780791	1	0.780791	F (1, 20) = 1.27947	P=0.271379	ns
PLX	19.6163	1	19.6163	F (1, 20) = 32.145	P=0.000015	****
Housing	6.1792	1	6.1792	F (1, 20) = 10.1258	P=0.004684	**
Residual	12.2049	20	0.610245			

Holm-Sidak adjustment	Individual P Value	1-(1-α)^(1/i) comparison	j, asc. rank	Adjusted P Value	Summary
PLX(-):SE vs. PLX(-):EE	0.162491	0.05	5	0.162491	ns
PLX(-):SE vs. PLX(+):SE	0.004403	0.016952428	3	0.013150926	*
PLX(-):SE vs. PLX(+):EE	0.000004	0.010206218	1	1.99998E-05	****
PLX(-):EE vs. PLX(+):SE	0.093875				
PLX(-):EE vs. PLX(+):EE	0.000107	0.012741455	2	0.000427931	***
PLX(+):SE vs. PLX(+):EE	0.006322	0.025320566	4	0.012604032	*

Cx3cr1

ANOVA table	SS	DF	MS	F (DFn, DFd)	P value	P value summary
Interaction	1.29371	1	1.29371	F (1, 20) = 1.50573	P=0.234041	ns
PLX	38.6178	1	38.6178	F (1, 20) = 44.9469	P=0.000002	****
Housing	1.03027	1	1.03027	F (1, 20) = 1.19913	P=0.286509	ns
Residual	17.1838	20	0.859188			

Holm-Sidak adjustment	Individual P Value	1-(1-α)^(1/i) comparison	j, asc. rank	Adjusted P Value	Summary
PLX(-):SE vs. PLX(-):EE	0.116223	0.025320566	4	0.218938214	ns
PLX(-):SE vs. PLX(+):SE	0.000017	0.010206218	1	8.49971E-05	****
PLX(-):SE vs. PLX(+):EE	0.000021	0.012741455	2	8.39974E-05	****
PLX(-):EE vs. PLX(+):SE	0.000761				
PLX(-):EE vs. PLX(+):EE	0.000947	0.016952428	3	0.00283831	**
PLX(+):SE vs. PLX(+):EE	0.926543	0.05	5	0.926543	ns

Nfkbia

ANOVA table	SS	DF	MS	F (DFn, DFd)	P value	P value summary
Interaction	0.0921006	1	0.0921006	F (1, 20) = 0.355519	P=0.557694	ns
PLX	0.179835	1	0.179835	F (1, 20) = 0.694183	P=0.414585	ns
Housing	28.9964	1	28.9964	F (1, 20) = 111.93	P<0.000001	****
Residual	5.18119	20	0.25906			

Holm-Sidak adjustment	Individual P Value	1-(1-α)^(1/i) comparison	j, asc. rank	Adjusted P Value	Summary
PLX(-):SE vs. PLX(-):EE	<0.000001	0.012741455	2	3.99999E-06	****
PLX(-):SE vs. PLX(+):SE	0.868636	0.05	5	0.868636	ns
PLX(-):SE vs. PLX(+):EE	0.000001	0.012741455	2	3.99999E-06	****
PLX(-):EE vs. PLX(+):SE	<0.000001				
PLX(-):EE vs. PLX(+):EE	0.324204	0.025320566	4	0.543299766	ns
PLX(+):SE vs. PLX(+):EE	<0.000001	0.012741455	2	3.99999E-06	****

Ikbkb

ANOVA table	SS	DF	MS	F (DFn, DFd)	P value	P value summary
Interaction	0.105241	1	0.105241	F (1, 20) = 0.896597	P=0.354997	ns
PLX	0.7091	1	0.7091	F (1, 20) = 6.04113	P=0.023226	*
Housing	5.30043	1	5.30043	F (1, 20) = 45.1567	P=0.000002	****
Residual	2.34757	20	0.117379			

Holm-Sidak adjustment	Individual P Value	1-(1-α)^(1/i) comparison	j, asc. rank	Adjusted P Value	Summary
PLX(-):SE vs. PLX(-):EE	0.000026	0.012741455	2	0.000103996	***
PLX(-):SE vs. PLX(+):SE	0.025836	0.025320566	4	0.051004501	ns
PLX(-):SE vs. PLX(+):EE	0.000003	0.010206218	1	1.49999E-05	****
PLX(-):EE vs. PLX(+):SE	0.006861				
PLX(-):EE vs. PLX(+):EE	0.298055	0.05	5	0.298055	ns
PLX(+):SE vs. PLX(+):EE	0.000581	0.016952428	3	0.001741988	**

Il1b

ANOVA table	SS	DF	MS	F (DFn, DFd)	P value	P value summary
Interaction	0.305488	1	0.305488	F (1, 20) = 0.318847	P=0.57858	ns
PLX	14.3746	1	14.3746	F (1, 20) = 15.0031	P=0.000946	***
Housing	3.33199	1	3.33199	F (1, 20) = 3.4777	P=0.076944	ns
Residual	19.1621	20	0.958104			

Holm-Sidak adjustment	Individual P Value	1-(1-α)^(1/i) comparison	j, asc. rank	Adjusted P Value	Summary
PLX(-):SE vs. PLX(-):EE	0.101254	0.025320566	4	0.192255627	ns
PLX(-):SE vs. PLX(+):SE	0.005176	0.012741455	2	0.020543808	*
PLX(-):SE vs. PLX(+):EE	0.000615	0.010206218	1	0.00307122	**
PLX(-):EE vs. PLX(+):SE	0.170938				
PLX(-):EE vs. PLX(+):EE	0.029789	0.016952428	3	0.086731281	ns
PLX(+):SE vs. PLX(+):EE	0.368856	0.05	5	0.368856	ns

Tgfb1

ANOVA table	SS	DF	MS	F (DFn, DFd)	P value	P value summary
Interaction	0.0815345	1	0.0815345	F (1, 20) = 0.308184	P=0.584953	ns
PLX	2.59621	1	2.59621	F (1, 20) = 9.81314	P=0.005242	**
Housing	5.48257	1	5.48257	F (1, 20) = 20.723	P=0.000194	***
Residual	5.29129	20	0.264565			

Holm-Sidak adjustment	Individual P Value	1-(1-α)^(1/i) comparison	j, asc. rank	Adjusted P Value	Summary
PLX(-):SE vs. PLX(-):EE	0.001741	0.012741455	2	0.006945835	**
PLX(-):SE vs. PLX(+):SE	0.016847	0.025320566	4	0.033410179	*
PLX(-):SE vs. PLX(+):EE	0.000026	0.010206218	1	0.000129993	***
PLX(-):EE vs. PLX(+):SE	0.327442				
PLX(-):EE vs. PLX(+):EE	0.083361	0.05	5	0.083361	ns
PLX(+):SE vs. PLX(+):EE	0.01043	0.016952428	3	0.03096478	*

Figure 5a

Adipose Tissue Macrophages

ANOVA table	SS (Type III)	DF	MS	F (DFn, DFd)	P value	P value summary
Interaction	0.723528	1	0.723528	F (1, 19) = 0.010117	P=0.920935	ns
PLX	30.9558	1	30.9558	F (1, 19) = 0.432851	P=0.518486	ns
Housing	9.32575	1	9.32575	F (1, 19) = 0.130401	P=0.722001	ns
Residual	1358.8	19	71.516			

Holm-Sidak adjustment	Individual P Value	1-(1-α)^(1/i) comparison	j, asc. rank	Adjusted P Value	Summary
PLX(-):SE vs. PLX(-):EE	0.741645	0.016952428	3	0.9827555	ns
PLX(-):SE vs. PLX(+):SE	0.690851	0.012741455	2	0.990865782	ns
PLX(-):SE vs. PLX(+):EE	0.839717	0.025320566	4	0.97430936	ns
PLX(-):EE vs. PLX(+):SE	0.469322				
PLX(-):EE vs. PLX(+):EE	0.606333	0.010206218	1	0.990545359	ns
PLX(+):SE vs. PLX(+):EE	0.85907	0.05	5	0.85907	ns

Figure 5b

ATM Polarization

CD11c+, CD206-

ANOVA table	SS (Type III)	DF	MS	F (DFn, DFd)	P value	P value summary
Interaction	9.19723	1	9.19723	F (1, 19) = 0.335975	P=0.568972	ns
PLX	51.7261	1	51.7261	F (1, 19) = 1.88956	P=0.185248	ns
Housing	43.2772	1	43.2772	F (1, 19) = 1.58092	P=0.223865	ns
Residual	520.12	19	27.3747			

Holm-Sidak adjustment	Individual P Value	1-(1-α)^(1/i) comparison	j, asc. rank	Adjusted P Value	Summary
PLX(-):SE vs. PLX(-):EE	0.629016	0.05	5	0.629016	ns
PLX(-):SE vs. PLX(+):SE	0.571365	0.025320566	4	0.816272037	ns
PLX(-):SE vs. PLX(+):EE	0.084822	0.010206218	1	0.358010599	ns
PLX(-):EE vs. PLX(+):SE	0.933176				
PLX(-):EE vs. PLX(+):EE	0.192852	0.012741455	2	0.575563524	ns
PLX(+):SE vs. PLX(+):EE	0.219741	0.016952428	3	0.524975116	ns

CD11c-, CD206+

ANOVA table	SS (Type III)	DF	MS	F (DFn, DFd)	P value	P value summary
Interaction	4.95647	1	4.95647	F (1, 19) = 1.09049	P=0.309468	ns
PLX	295.242	1	295.242	F (1, 19) = 64.9572	P<0.000001	****
Housing	4.34505	1	4.34505	F (1, 19) = 0.955969	P=0.34049	ns
Residual	86.3584	19	4.54518			

Holm-Sidak adjustment	Individual P Value	1-(1-α)^(1/i) comparison	j, asc. rank	Adjusted P Value	Summary
PLX(-):SE vs. PLX(-):EE	0.159252	0.025320566	4	0.2931428	ns
PLX(-):SE vs. PLX(+):SE	0.000066	0.012741455	2	0.000263974	***
PLX(-):SE vs. PLX(+):EE	0.000101	0.016952428	3	0.000302969	***
PLX(-):EE vs. PLX(+):SE	0.000003				
PLX(-):EE vs. PLX(+):EE	0.000005	0.010206218	1	2.49998E-05	****
PLX(+):SE vs. PLX(+):EE	0.963821	0.05	5	0.963821	ns

CD11c+, CD206+

ANOVA table	SS (Type III)	DF	MS	F (DFn, DFd)	P value	P value summary
Interaction	42.4062	1	42.4062	F (1, 19) = 0.521636	P=0.47894	ns
PLX	2481.15	1	2481.15	F (1, 19) = 30.5205	P=0.000025	****
Housing	162.083	1	162.083	F (1, 19) = 1.99377	P=0.174116	ns
Residual	1544.6	19	81.2946			

Holm-Sidak adjustment	Individual P Value	1-(1-α)^(1/i) comparison	j, asc. rank	Adjusted P Value	Summary
PLX(-):SE vs. PLX(-):EE	0.138499	0.025320566	4	0.257816027	ns
PLX(-):SE vs. PLX(+):SE	0.000231	0.012741455	2	0.00092368	***
PLX(-):SE vs. PLX(+):EE	0.000127	0.010206218	1	0.000634839	***
PLX(-):EE vs. PLX(+):SE	0.007698				
PLX(-):EE vs. PLX(+):EE	0.003618	0.016952428	3	0.010814778	*
PLX(+):SE vs. PLX(+):EE	0.639135	0.05	5	0.639135	ns

Figure 6

rWAT qRT-PCR

Leptin

ANOVA table	SS (Type III)	DF	MS	F (DFn, DFd)	P value	P value summary
Interaction	0.225501	1	0.225501	F (1, 18) = 0.625739	P=0.439222	ns
PLX	1.39574	1	1.39574	F (1, 18) = 3.87301	P=0.064669	ns
Housing	3.38075	1	3.38075	F (1, 18) = 9.3812	P=0.006702	**
Residual	6.48676	18	0.360376			

Holm-Sidak adjustment	Individual P Value	1-(1-α)^(1/i) comparison	j, asc. rank	Adjusted P Value	Summary
PLX(-):SE vs. PLX(-):EE	0.109279	0.025320566	4	0.2066161	ns
PLX(-):SE vs. PLX(+):SE	0.416181	0.05	5	0.416181	ns
PLX(-):SE vs. PLX(+):EE	0.002251	0.010206218	1	0.011204444	*
PLX(-):EE vs. PLX(+):SE	0.448866				
PLX(-):EE vs. PLX(+):EE	0.066809	0.016952428	3	0.187334871	ns
PLX(+):SE vs. PLX(+):EE	0.017756	0.012741455	2	0.06915464	ns

Adrb3

ANOVA table	SS (Type III)	DF	MS	F (DFn, DFd)	P value	P value summary
Interaction	0.172	1	0.172	F (1, 18) = 0.61052	P=0.444748	ns
PLX	4.46438	1	4.46438	F (1, 18) = 15.8465	P=0.000877	***
Housing	0.0676389	1	0.0676389	F (1, 18) = 0.240087	P=0.630062	ns
Residual	5.07109	18	0.281727			

Holm-Sidak adjustment	Individual P Value	1-(1-α)^(1/i) comparison	j, asc. rank	Adjusted P Value	Summary
PLX(-):SE vs. PLX(-):EE	0.83135	0.05	5	0.83135	ns
PLX(-):SE vs. PLX(+):SE	0.036285	0.016952428	3	0.104952969	ns
PLX(-):SE vs. PLX(+):EE	0.005403	0.012741455	2	0.021437476	*
PLX(-):EE vs. PLX(+):SE	0.023822				
PLX(-):EE vs. PLX(+):EE	0.003431	0.010206218	1	0.017037686	*
PLX(+):SE vs. PLX(+):EE	0.400721	0.025320566	4	0.64086468	ns

Hsl

ANOVA table	SS (Type III)	DF	MS	F (DFn, DFd)	P value	P value summary
Interaction	5.59721E-05	1	5.59721E-05	F (1, 18) = 0.000165056	P=0.989891	ns
PLX	2.20283	1	2.20283	F (1, 18) = 6.4959	P=0.020154	*
Housing	0.305459	1	0.305459	F (1, 18) = 0.900765	P=0.355146	ns
Residual	6.10399	18	0.339111			

Holm-Sidak adjustment	Individual P Value	1-(1-α)^(1/i) comparison	j, asc. rank	Adjusted P Value	Summary
PLX(-):SE vs. PLX(-):EE	0.484752	0.025320566	4	0.734519498	ns
PLX(-):SE vs. PLX(+):SE	0.086817	0.012741455	2	0.304605467	ns
PLX(-):SE vs. PLX(+):EE	0.023578	0.010206218	1	0.112460317	ns
PLX(-):EE vs. PLX(+):SE	0.272858				
PLX(-):EE vs. PLX(+):EE	0.089774	0.016952428	3	0.245867409	ns
PLX(+):SE vs. PLX(+):EE	0.53416	0.05	5	0.53416	ns

Srebp1c

ANOVA table	SS (Type III)	DF	MS	F (DFn, DFd)	P value	P value summary
Interaction	0.135534	1	0.135534	F (1, 18) = 0.458906	P=0.50675	ns
PLX	3.61807	1	3.61807	F (1, 18) = 12.2505	P=0.002556	**
Housing	0.00352515	1	0.00352515	F (1, 18) = 0.0119359	P=0.914212	ns
Residual	5.31614	18	0.295341			

Holm-Sidak adjustment	Individual P Value	1-(1-α)^(1/i) comparison	j, asc. rank	Adjusted P Value	Summary
PLX(-):SE vs. PLX(-):EE	0.678474	0.05	5	0.678474	ns
PLX(-):SE vs. PLX(+):SE	0.008494	0.010206218	1	0.041754622	*
PLX(-):SE vs. PLX(+):EE	0.027558	0.012741455	2	0.105758478	ns
PLX(-):EE vs. PLX(+):SE	0.020009				
PLX(-):EE vs. PLX(+):EE	0.061302	0.016952428	3	0.172862563	ns
PLX(+):SE vs. PLX(+):EE	0.600836	0.025320566	4	0.840668101	ns

Pparg

ANOVA table	SS (Type III)	DF	MS	F (DFn, DFd)	P value	P value summary
Interaction	0.0502365	1	0.0502365	F (1, 18) = 0.235154	P=0.633577	ns
PLX	1.18553	1	1.18553	F (1, 18) = 5.54943	P=0.030028	*
Housing	0.0328992	1	0.0328992	F (1, 18) = 0.153999	P=0.69935	ns
Residual	3.84537	18	0.213632			

Holm-Sidak adjustment	Individual P Value	1-(1-α)^(1/i) comparison	j, asc. rank	Adjusted P Value	Summary
PLX(-):SE vs. PLX(-):EE	0.523483	0.025320566	4	0.772931549	ns
PLX(-):SE vs. PLX(+):SE	0.059818	0.010206218	1	0.265385221	ns
PLX(-):SE vs. PLX(+):EE	0.067794	0.012741455	2	0.24482505	ns
PLX(-):EE vs. PLX(+):SE	0.182002				
PLX(-):EE vs. PLX(+):EE	0.202446	0.016952428	3	0.492681976	ns
PLX(+):SE vs. PLX(+):EE	0.950757	0.05	5	0.950757	ns

Ppargc1a

ANOVA table	SS (Type III)	DF	MS	F (DFn, DFd)	P value	P value summary
Interaction	1.62384	1	1.62384	F (1, 18) = 2.25978	P=0.150116	ns
PLX	3.28678	1	3.28678	F (1, 18) = 4.57397	P=0.046423	*
Housing	4.70858	1	4.70858	F (1, 18) = 6.55258	P=0.019692	*
Residual	12.9345	18	0.718585			

Holm-Sidak adjustment	Individual P Value	1-(1-α)^(1/i) comparison	j, asc. rank	Adjusted P Value	Summary
PLX(-):SE vs. PLX(-):EE	0.007467	0.012741455	2	0.029535126	*
PLX(-):SE vs. PLX(+):SE	0.019065	0.016952428	3	0.056111507	ns
PLX(-):SE vs. PLX(+):EE	0.00379	0.010206218	1	0.018806902	*
PLX(-):EE vs. PLX(+):SE	0.769285				
PLX(-):EE vs. PLX(+):EE	0.658567	0.05	5	0.658567	ns
PLX(+):SE vs. PLX(+):EE	0.483609	0.025320566	4	0.733340335	ns

Il1b

ANOVA table	SS (Type III)	DF	MS	F (DFn, DFd)	P value	P value summary
Interaction	0.0651929	1	0.0651929	F (1, 18) = 0.0680206	P=0.797201	ns
PLX	0.108958	1	0.108958	F (1, 18) = 0.113684	P=0.739888	ns
Housing	2.80497	1	2.80497	F (1, 18) = 2.92664	P=0.104308	ns
Residual	17.2517	18	0.958428			

Holm-Sidak adjustment	Individual P Value	1-(1-α)^(1/i) comparison	j, asc. rank	Adjusted P Value	Summary
PLX(-):SE vs. PLX(-):EE	0.296443	0.012741455	2	0.754982472	ns
PLX(-):SE vs. PLX(+):SE	0.677424	0.025320566	4	0.895944724	ns
PLX(-):SE vs. PLX(+):EE	0.34429	0.016952428	3	0.718073811	ns
PLX(-):EE vs. PLX(+):SE	0.164786				
PLX(-):EE vs. PLX(+):EE	0.957532	0.05	5	0.957532	ns
PLX(+):SE vs. PLX(+):EE	0.198598	0.010206218	1	0.669438622	ns

Ccl2

ANOVA table	SS (Type III)	DF	MS	F (DFn, DFd)	P value	P value summary
Interaction	1.53761	1	1.53761	F (1, 18) = 2.03431	P=0.170899	ns
PLX	0.091947	1	0.091947	F (1, 18) = 0.121649	P=0.731299	ns
Housing	1.48995	1	1.48995	F (1, 18) = 1.97125	P=0.177334	ns
Residual	13.6051	18	0.755838			

Holm-Sidak adjustment	Individual P Value	1-(1-α)^(1/i) comparison	j, asc. rank	Adjusted P Value	Summary
PLX(-):SE vs. PLX(-):EE	0.986999	0.05	5	0.986999	ns
PLX(-):SE vs. PLX(+):SE	0.22547	0.012741455	2	0.640123924	ns
PLX(-):SE vs. PLX(+):EE	0.465204	0.025320566	4	0.713993238	ns
PLX(-):EE vs. PLX(+):SE	0.23111				
PLX(-):EE vs. PLX(+):EE	0.455979	0.016952428	3	0.838992171	ns
PLX(+):SE vs. PLX(+):EE	0.071369	0.010206218	1	0.309416994	ns

Supplementary Figure 1a

Body Weight

ANOVA table	SS	DF	MS	F (DFn, DFd)	P value	P value summary
Interaction	4.99509	10	0.499509	F (10, 180) = 1.70962	P=0.081499	ns
Time	255.891	10	25.5891	F (10, 180) = 87.5813	P<0.000001	****
PLX	9.82841	1	9.82841	F (1, 18) = 0.546017	P=0.469476	ns
Subjects (matching)	324.003	18	18.0002	F (18, 180) = 61.6074	P<0.000001	****
Residual	52.5916	180	0.292176			

Holm-Sidak's multiple comparisons test

PLX(-) - PLX(+)	Individual P Value	1-(1-α)^(1/i) comparison	j, asc. rank	Adjusted P Value	Summary
Row 1	0.948355	0.025320566	10	0.997332794	ns
Row 2	0.98708	0.05	11	0.999999	ns
Row 3	0.833278	0.016952428	9	0.995365758	ns
Row 4	0.486504	0.008512445	6	0.981667471	ns
Row 5	0.615788	0.012741455	8	0.978208672	ns
Row 6	0.331831	0.005683045	3	0.973455356	ns
Row 7	0.339934	0.006391151	4	0.963967126	ns
Row 8	0.400185	0.007300832	5	0.972066764	ns
Row 9	0.593216	0.010206218	7	0.98886169	ns
Row 10	0.121184	0.004652172	1	0.758522035	ns
Row 11	0.1906	0.005116197	2	0.879320917	ns

Supplementary Figure 1b

Body Weight, % Change

ANOVA table	SS	DF	MS	F (DFn, DFd)	P value	P value summary
Interaction	157.602	10	15.7602	F (10, 180) = 1.88041	P=0.050462	ns
Time	7403.46	10	740.346	F (10, 180) = 88.3333	P<0.000001	****
PLX	311.804	1	311.804	F (1, 18) = 2.83516	P=0.109483	ns
Subjects (matching)	1979.59	18	109.977	F (18, 180) = 13.1218	P<0.000001	****
Residual	1508.63	180	8.38128			

Holm-Sidak's multiple comparisons test

PLX(-) - PLX(+)	Individual P Value	1-(1-α)^(1/i) comparison	j, asc. rank	Adjusted P Value	Summary
Row 1	0.899655	0.05	10	0.899655	ns
Row 2	>0.999999				
Row 3	0.659231	0.025320566	9	0.883876489	ns
Row 4	0.192973	0.010206218	6	0.657673654	ns
Row 5	0.33903	0.012741455	7	0.809134694	ns
Row 6	0.080924	0.007300832	4	0.446063506	ns
Row 7	0.080832	0.006391151	3	0.490482423	ns
Row 8	0.110525	0.008512445	5	0.504775096	ns
Row 9	0.346584	0.016952428	8	0.721022425	ns
Row 10	0.005219	0.005116197	1	0.050981196	ns
Row 11	0.013498	0.005683045	2	0.115125397	ns

Supplementary Figure 1c

Relative Food Intake

Unpaired t test with Welch's correction	
P value	0.84492
P value summary	ns
Significantly different (P < 0.05)?	No
One- or two-tailed P value?	Two-tailed
Welch-corrected t, df	t=0.197315 df=29.8192
F test to compare variances	
F, DFn, Dfd	1.16888, 15, 15
P value	0.766431
P value summary	ns
Significantly different (P < 0.05)?	No

Supplementary Figure 1d

Body Fat %

Unpaired t test with Welch's correction	
P value	0.972094
P value summary	ns
Significantly different (P < 0.05)?	No
One- or two-tailed P value?	Two-tailed
Welch-corrected t, df	t=0.0355321 df=16.0533
F test to compare variances	
F, DFn, Dfd	2.06858, 9, 9
P value	0.293958
P value summary	ns
Significantly different (P < 0.05)?	No

Supplementary Figure 1e

Lean Weight %

Unpaired t test with Welch's correction	
P value	0.9585
P value summary	ns
Significantly different (P < 0.05)?	No
One- or two-tailed P value?	Two-tailed
Welch-corrected t, df	t=0.0527809 df=17.6387
F test to compare variances	
F, DFn, Dfd	1.33403, 9, 9
P value	0.674644
P value summary	ns
Significantly different (P < 0.05)?	No

Supplementary Figure 1f

Glucose Tolerance Test, RM

ANOVA table	SS	DF	MS	F (DFn, DFd)	P value	P value summary
Interaction	15296.5	5	3059.29	F (5, 90) = 4.21192	P=0.001741	**
Time	962259	5	192452	F (5, 90) = 264.961	P<0.000001	****
Diet	2842.13	1	2842.13	F (1, 18) = 1.09236	P=0.309783	ns
Subjects (matching)	46833.1	18	2601.84	F (18, 90) = 3.58211	P=0.000029	****
Residual	65370.7	90	726.341			

Holm-Sidak's multiple comparisons test

PLX(-) - PLX(+)	Individual P Value	1-(1-α)^(1/i) comparison	j, asc. rank	Adjusted P Value	Summary
Row 1	0.22215	0.010206218	2	0.715239806	ns
Row 2	0.000186	0.008512445	1	0.001115481	**
Row 3	0.797913	0.025320566	5	0.959160844	ns
Row 4	0.529179	0.016952428	4	0.895631972	ns
Row 5	0.480712	0.012741455	3	0.92728347	ns
Row 6	0.972394	0.05	6	0.972394	ns

Row 1: Fasting Blood Glucose

Supplementary Figure 1g

Glucose Tolerance Test, AUC

Unpaired t test with Welch's correction	
P value	0.692366
P value summary	ns
Significantly different (P < 0.05)?	No
One- or two-tailed P value?	Two-tailed
Welch-corrected t, df	t=0.402501 df=16.8817

F test to compare variances	
F, DFn, Dfd	1.69314, 9, 9
P value	0.444846
P value summary	ns
Significantly different (P < 0.05)?	No

Supplementary Figure 1h

Relative Tissue Weights at Sacrifice

BAT

Unpaired t test with Welch's correction	
P value	0.86124
P value summary	ns
Significantly different (P < 0.05)?	No
One- or two-tailed P value?	Two-tailed
Welch-corrected t, df	t=0.179213 df=10.3202
F test to compare variances	
F, DFn, Dfd	2.35261, 6, 6
P value	0.32161
P value summary	ns
Significantly different (P < 0.05)?	No

iWAT

Unpaired t test with Welch's correction	
P value	0.409036
P value summary	ns
Significantly different (P < 0.05)?	No
One- or two-tailed P value?	Two-tailed
Welch-corrected t, df	t=0.864028 df=9.41928
F test to compare variances	
F, DFn, Dfd	3.19668, 6, 6
P value	0.183092
P value summary	ns
Significantly different (P < 0.05)?	No

gWAT

Unpaired t test with Welch's correction	
P value	0.278114
P value summary	ns
Significantly different (P < 0.05)?	No
One- or two-tailed P value?	Two-tailed
Welch-corrected t, df	t=1.14357 df=10.5385
F test to compare variances	
F, DFn, Dfd	2.18676, 6, 6
P value	0.363617
P value summary	ns
Significantly different (P < 0.05)?	No

rWAT

Unpaired t test with Welch's correction	
P value	0.082193
P value summary	ns
Significantly different (P < 0.05)?	No
One- or two-tailed P value?	Two-tailed
Welch-corrected t, df	t=1.98523 df=8.04161
F test to compare variances	
F, DFn, Dfd	5.70235, 6, 6
P value	0.052448
P value summary	ns
Significantly different (P < 0.05)?	No

Liver

Unpaired t test with Welch's correction	
P value	0.513654
P value summary	ns
Significantly different (P < 0.05)?	No
One- or two-tailed P value?	Two-tailed
Welch-corrected t, df	t=0.675107 df=10.9161
F test to compare variances	
F, DFn, Dfd	1.92021, 6, 6
P value	0.447104
P value summary	ns
Significantly different (P < 0.05)?	No

Soleus

Unpaired t test with Welch's correction	
P value	0.504978
P value summary	ns
Significantly different (P < 0.05)?	No
One- or two-tailed P value?	Two-tailed
Welch-corrected t, df	t=0.689185 df=11.0051
F test to compare variances	
F, DFn, Dfd	1.85986, 6, 6
P value	0.469306
P value summary	ns
Significantly different (P < 0.05)?	No

Gastrocnemius

Unpaired t test with Welch's correction	
P value	0.000324
P value summary	***
Significantly different (P < 0.05)?	Yes
One- or two-tailed P value?	Two-tailed
Welch-corrected t, df	t=5.0676 df=11.4035
F test to compare variances	
F, DFn, Dfd	1.59304, 6, 6
P value	0.585896
P value summary	ns
Significantly different (P < 0.05)?	No

Spleen

Unpaired t test with Welch's correction	
P value	0.009876
P value summary	**
Significantly different (P < 0.05)?	Yes
One- or two-tailed P value?	Two-tailed
Welch-corrected t, df	t=3.0663 df=11.8925
F test to compare variances	
F, DFn, Dfd	1.21017, 6, 6
P value	0.822777
P value summary	ns
Significantly different (P < 0.05)?	No

Supplementary Figure 2

Hypothalamic qRT-PCR

Il1b

Unpaired t test with Welch's correction	
P value	0.016775
P value summary	*
Significantly different (P < 0.05)?	Yes
One- or two-tailed P value?	Two-tailed
Welch-corrected t, df	t=3.22792, df=6.29892
F test to compare variances	
F, DFn, Dfd	7.56654, 5, 5
P value	0.044404
P value summary	*
Significantly different (P < 0.05)?	Yes

Ly6d

Unpaired t test with Welch's correction	
P value	0.00056
P value summary	***
Significantly different (P < 0.05)?	Yes
One- or two-tailed P value?	Two-tailed
Welch-corrected t, df	t=5.06712, df=9.55752
F test to compare variances	
F, DFn, Dfd	1.54831, 5, 5
P value	0.643119
P value summary	ns
Significantly different (P < 0.05)?	No

H2Ab1

Unpaired t test with Welch's correction	
P value	0.166216
P value summary	ns
Significantly different (P < 0.05)?	No
One- or two-tailed P value?	Two-tailed
Welch-corrected t, df	t=1.60622, df=5.26306
F test to compare variances	
F, DFn, Dfd	32.6361, 5, 6
P value	0.000562
P value summary	***
Significantly different (P < 0.05)?	Yes

Supplementary Figure 5a

Red Pulp Macrophages

ANOVA table	SS (Type III)	DF	MS	F (DFn, DFd)	P value	P value summary
Interaction	1.81073	1	1.81073	F (1, 18) = 2.40124	P=0.138642	ns
PLX	10.2651	1	10.2651	F (1, 18) = 13.6126	P=0.001677	**
Housing	0.196564	1	0.196564	F (1, 18) = 0.260666	P=0.615867	ns
Residual	13.5735	18	0.754082			

Holm-Sidak adjustment	Individual P Value	1-(1-α)^(1/i) comparison	j, asc. rank	Adjusted P Value	Summary
PLX(-):SE vs. PLX(-):EE	0.16241	0.025320566	4	0.298442992	ns
PLX(-):SE vs. PLX(+):SE	0.147598	0.016952428	3	0.380653939	ns
PLX(-):SE vs. PLX(+):EE	0.045202	0.012741455	2	0.168913931	ns
PLX(-):EE vs. PLX(+):SE	0.005982				
PLX(-):EE vs. PLX(+):EE	0.001622	0.010206218	1	0.008083734	**
PLX(+):SE vs. PLX(+):EE	0.471979	0.05	5	0.471979	ns

Douple Positive Macrophages

ANOVA table	SS (Type III)	DF	MS	F (DFn, DFd)	P value	P value summary
Interaction	0.00288545	1	0.00288545	F (1, 18) = 0.00173324	P=0.96725	ns
PLX	17.0692	1	17.0692	F (1, 18) = 10.2532	P=0.00494	**
Housing	0.702987	1	0.702987	F (1, 18) = 0.422271	P=0.524013	ns
Residual	29.966	18	1.66478			

Holm-Sidak adjustment	Individual P Value	1-(1-α)^(1/i) comparison	j, asc. rank	Adjusted P Value	Summary
PLX(-):SE vs. PLX(-):EE	0.672259	0.05	5	0.672259	ns
PLX(-):SE vs. PLX(+):SE	0.03835	0.016952428	3	0.110694235	ns
PLX(-):SE vs. PLX(+):EE	0.017807	0.010206218	1	0.085920071	ns
PLX(-):EE vs. PLX(+):SE	0.07458				
PLX(-):EE vs. PLX(+):EE	0.034064	0.012741455	2	0.129450622	ns
PLX(+):SE vs. PLX(+):EE	0.630793	0.025320566	4	0.863686191	ns

Supplementary Figure 5b

Ly6Cint Monocytes

ANOVA table	SS (Type III)	DF	MS	F (DFn, DFd)	P value	P value summary
Interaction	0.0654014	1	0.0654014	F (1, 18) = 1.00414	P=0.329594	ns
PLX	0.69064	1	0.69064	F (1, 18) = 10.6037	P=0.004384	**
Housing	0.0731856	1	0.0731856	F (1, 18) = 1.12365	P=0.303151	ns
Residual	1.17238	18	0.065132			

Holm-Sidak adjustment	Individual P Value	1-(1-α)^(1/i) comparison	j, asc. rank	Adjusted P Value	Summary
PLX(-):SE vs. PLX(-):EE	0.162036	0.025320566	4	0.297816335	ns
PLX(-):SE vs. PLX(+):SE	0.128342	0.012741455	2	0.422722698	ns
PLX(-):SE vs. PLX(+):EE	0.154347	0.016952428	3	0.395249019	ns
PLX(-):EE vs. PLX(+):SE	0.004951				
PLX(-):EE vs. PLX(+):EE	0.007501	0.010206218	1	0.036946555	*
PLX(+):SE vs. PLX(+):EE	0.967761	0.05	5	0.967761	ns

Ly6Chi Monocytes

ANOVA table	SS (Type III)	DF	MS	F (DFn, DFd)	P value	P value summary
Interaction	4.09855	1	4.09855	F (1, 18) = 1.69469	P=0.209398	ns
PLX	10.9392	1	10.9392	F (1, 18) = 4.52323	P=0.047524	*
Housing	10.8109	1	10.8109	F (1, 18) = 4.47015	P=0.048707	*
Residual	43.5323	18	2.41846			

Holm-Sidak adjustment	Individual P Value	1-(1-α)^(1/i) comparison	j, asc. rank	Adjusted P Value	Summary
PLX(-):SE vs. PLX(-):EE	0.572738	0.05	5	0.572738	ns
PLX(-):SE vs. PLX(+):SE	0.566894	0.025320566	4	0.812419193	ns
PLX(-):SE vs. PLX(+):EE	0.010157	0.010206218	1	0.049763779	*
PLX(-):EE vs. PLX(+):SE	0.992697				
PLX(-):EE vs. PLX(+):EE	0.026086	0.012741455	2	0.100331665	ns
PLX(+):SE vs. PLX(+):EE	0.026565	0.016952428	3	0.077596649	ns

Supplementary Figure 5c

% CSF1R+ of

Red Pulp Macrophages

ANOVA table	SS (Type III)	DF	MS	F (DFn, DFd)	P value	P value summary
Interaction	0.773194	1	0.773194	F (1, 18) = 0.0980541	P=0.757777	ns
PLX	9.46324	1	9.46324	F (1, 18) = 1.2001	P=0.287744	ns
Housing	1.8423	1	1.8423	F (1, 18) = 0.233635	P=0.634669	ns
Residual	141.937	18	7.88538			

Holm-Sidak adjustment	Individual P Value	1-(1-α)^(1/i) comparison	j, asc. rank	Adjusted P Value	Summary
PLX(-):SE vs. PLX(-):EE	0.905527	0.05	5	0.905527	ns
PLX(-):SE vs. PLX(+):SE	0.332428	0.010206218	1	0.867416169	ns
PLX(-):SE vs. PLX(+):EE	0.683466	0.025320566	4	0.899806227	ns
PLX(-):EE vs. PLX(+):SE	0.256905				
PLX(-):EE vs. PLX(+):EE	0.586927	0.016952428	3	0.929517642	ns
PLX(+):SE vs. PLX(+):EE	0.580243	0.012741455	2	0.968954991	ns

Douple Positive Macrophages

ANOVA table	SS (Type III)	DF	MS	F (DFn, DFd)	P value	P value summary
Interaction	0.0305456	1	0.0305456	F (1, 18) = 0.00762539	P=0.931379	ns
PLX	1.9278	1	1.9278	F (1, 18) = 0.481255	P=0.49671	ns
Housing	26.6526	1	26.6526	F (1, 18) = 6.65354	P=0.018898	*
Residual	72.104	18	4.00578			

Holm-Sidak adjustment	Individual P Value	1-(1-α)^(1/i) comparison	j, asc. rank	Adjusted P Value	Summary
PLX(-):SE vs. PLX(-):EE	0.075582	0.010206218	1	0.324940635	ns
PLX(-):SE vs. PLX(+):SE	0.673163	0.05	5	0.673163	ns
PLX(-):SE vs. PLX(+):EE	0.217955	0.016952428	3	0.521705672	ns
PLX(-):EE vs. PLX(+):SE	0.025922				
PLX(-):EE vs. PLX(+):EE	0.587546	0.025320566	4	0.829881698	ns
PLX(+):SE vs. PLX(+):EE	0.095009	0.012741455	2	0.329224733	ns

Ly6Cint Monocytes

ANOVA table	SS (Type III)	DF	MS	F (DFn, DFd)	P value	P value summary
Interaction	3.30721	1	3.30721	F (1, 18) = 0.626208	P=0.439053	ns
PLX	0.0244855	1	0.0244855	F (1, 18) = 0.00463623	P=0.946465	ns
Housing	37.6425	1	37.6425	F (1, 18) = 7.12747	P=0.015623	*
Residual	95.0639	18	5.28133			

Holm-Sidak adjustment	Individual P Value	1-(1-α)^(1/i) comparison	j, asc. rank	Adjusted P Value	Summary
PLX(-):SE vs. PLX(-):EE	0.200699	0.016952428	3	0.489340908	ns
PLX(-):SE vs. PLX(+):SE	0.61528	0.05	5	0.61528	ns
PLX(-):SE vs. PLX(+):EE	0.080273	0.012741455	2	0.284456989	ns
PLX(-):EE vs. PLX(+):SE	0.069594				
PLX(-):EE vs. PLX(+):EE	0.550975	0.025320566	4	0.798376549	ns
PLX(+):SE vs. PLX(+):EE	0.02488	0.010206218	1	0.11836196	ns

Ly6Chi Monocytes

ANOVA table	SS (Type III)	DF	MS	F (DFn, DFd)	P value	P value summary
Interaction	7.42	1	7.42	F (1, 18) = 1.12935	P=0.301963	ns
PLX	4.04044	1	4.04044	F (1, 18) = 0.614971	P=0.44312	ns
Housing	30.311	1	30.311	F (1, 18) = 4.61346	P=0.045586	*
Residual	118.262	18	6.57012			

Holm-Sidak adjustment	Individual P Value	1-(1-α)^(1/i) comparison	j, asc. rank	Adjusted P Value	Summary
PLX(-):SE vs. PLX(-):EE	0.452826	0.025320566	4	0.700600614	ns
PLX(-):SE vs. PLX(+):SE	0.846084	0.05	5	0.846084	ns
PLX(-):SE vs. PLX(+):EE	0.062594	0.012741455	2	0.227833572	ns
PLX(-):EE vs. PLX(+):SE	0.325254				
PLX(-):EE vs. PLX(+):EE	0.208009	0.016952428	3	0.503223848	ns
PLX(+):SE vs. PLX(+):EE	0.035711	0.010206218	1	0.166249585	ns

Supplementary Figure 5d

Splenic DP Macrophage Polarization

CD11c+, CD206-

ANOVA table	SS (Type III)	DF	MS	F (DFn, DFd)	P value	P value summary
Interaction	5.41824	1	5.41824	F (1, 18) = 0.213413	P=0.64964	ns
PLX	140.578	1	140.578	F (1, 18) = 5.53705	P=0.030189	*
Housing	0.693879	1	0.693879	F (1, 18) = 0.0273304	P=0.870535	ns
Residual	456.993	18	25.3885			

Holm-Sidak adjustment	Individual P Value	1-(1-α)^(1/i) comparison	j, asc. rank	Adjusted P Value	Summary
PLX(-):SE vs. PLX(-):EE	0.83621	0.05	5	0.83621	ns
PLX(-):SE vs. PLX(+):SE	0.061936	0.010206218	1	0.27362256	ns
PLX(-):SE vs. PLX(+):EE	0.155865	0.012741455	2	0.492252691	ns
PLX(-):EE vs. PLX(+):SE	0.078173				
PLX(-):EE vs. PLX(+):EE	0.197802	0.016952428	3	0.483768234	ns
PLX(+):SE vs. PLX(+):EE	0.662648	0.025320566	4	0.886193628	ns

CD11c-, CD206+

ANOVA table	SS (Type III)	DF	MS	F (DFn, DFd)	P value	P value summary
Interaction	0.0563788	1	0.0563788	F (1, 18) = 0.816711	P=0.37808	ns
PLX	0.458727	1	0.458727	F (1, 18) = 6.64519	P=0.018962	*
Housing	0.00490909	1	0.00490909	F (1, 18) = 0.0711138	P=0.792753	ns
Residual	1.24257	18	0.0690315			

Holm-Sidak adjustment	Individual P Value	1-(1-α)^(1/i) comparison	j, asc. rank	Adjusted P Value	Summary
PLX(-):SE vs. PLX(-):EE	0.657756	0.05	5	0.657756	ns
PLX(-):SE vs. PLX(+):SE	0.024146	0.010206218	1	0.115038794	ns
PLX(-):SE vs. PLX(+):EE	0.135073	0.012741455	2	0.440348312	ns
PLX(-):EE vs. PLX(+):SE	0.049163				
PLX(-):EE vs. PLX(+):EE	0.251901	0.016952428	3	0.581324813	ns
PLX(+):SE vs. PLX(+):EE	0.418739	0.025320566	4	0.66213565	ns
